# Comprehensive Annotation of Mature Peptides and Genotypes for Zika Virus

**DOI:** 10.1371/journal.pone.0170462

**Published:** 2017-01-26

**Authors:** Guangyu Sun, Christopher N. Larsen, Nicole Baumgarth, Edward B. Klem, Richard H. Scheuermann

**Affiliations:** 1 Vecna Technologies, Inc., Greenbelt, Maryland, United States of America; 2 Center for Comparative Medicine and the Department of Pathology, Microbiology & Immunology, University of California, Davis, Davis, California, United States of America; 3 Northrop Grumman Health Solutions, Rockville, Maryland, United States of America; 4 J. Craig Venter Institute, La Jolla, California, United States of America; 5 Department of Pathology, University of California, San Diego, San Diego, California, United States of America; 6 Division of Vaccine Discovery, La Jolla Institute for Allergy and Immunology, La Jolla, California, United States of America; SRI International, UNITED STATES

## Abstract

The rapid spread of Zika virus (ZIKV) has caused much concern in the global health community, due in part to a link to fetal microcephaly and other neurological illnesses. While an increasing amount of ZIKV genomic sequence data is being generated, an understanding of the virus molecular biology is still greatly lacking. A significant step towards establishing ZIKV proteomics would be the compilation of all proteins produced by the virus, and the resultant virus genotypes. Here we report for the first time such data, using new computational methods for the annotation of mature peptide proteins, genotypes, and recombination events for all ZIKV genomes. The data is made publicly available through the Virus Pathogen Resource at www.viprbrc.org.

## Introduction

The recent emergence of Zika virus (ZIKV) poses a global public health threat [1]. ZIKV is a mosquito-borne arbovirus of the *Flaviviridae* family, first isolated from a Rhesus macaque monkey in the Zika forest of Uganda in 1947. Until recently ZIKV outbreaks had only been seen in Africa, Asia, and the Pacific Islands [2]. Zika’s epidemiologic range has now expanded to Brazil and other South, Central and North American countries. In most cases, symptomatic patients experience mild fever, rash, and arthralgia. However, the virus has evolved, and new and alarming correlations between ZIKV infection and microcephaly and Guillian-Barre syndrome now imply a causal relationship between infection and birth defects.

ZIKV has become global, yet there are no vaccines or antivirals available, and only one differential diagnostic test is available at the CDC to distinguish ZIKV from co-endemic Dengue and Chikungunya viruses [3]. The Trioplex Real-Time RT-PCR (rRT-PCR) assay and the Zika MAC-ELISA were approved by the FDA under Emergency Use Authorization, and are being distributed to qualified laboratories. The Zika MAC test is known to cross-react with other flaviviruses, but can be used in the context of anti-IgM DENV, Chikungunya, and ZIKV tests. However the serology of these diagnostics and cross reactions is not well understood at the virus protein level.

Therefore to gain a better understanding of ZIKV, for generating vaccines and diagnostics, and for advancing our knowledge on the epidemiology, ecology, and pathogenesis of this virus, we must first enhance our understanding of ZIKV’s genome and protein sequences, protein structures, and relevant metadata. There is urgent need because currently only a quarter of the available ZIKV genomes in public repositories include any protein annotation other than the polyprotein, and few experimental laboratory data for ZIKV exist. Hopefully in time the increased awareness brought on by ongoing outbreaks will increase the availability of genomic data sets. Nonetheless, incomplete and inconsistent annotation of currently available ZIKV genomes and proteomes makes it difficult to even assess the virus protein biology, much less integrate any new or existing protein data. The absence of data is negatively impacting research progress, and could delay the development of peptide vaccine candidates, diagnostics, and therapies. We posit that a well described, modern, and consistent ZIKV protein annotation method is needed that is based on reference sequences and known standards, and that, when applied universally to all Zika genomes, can produce a complete set of proteins that can be viewed in the context of their genotype and genome information.

Described here is a new set of data and sequence analysis tools for the full and rapid annotation of all available ZIKV genome sequence data. Advances of this work include the i) computational identification of polyprotein maturation cleavage sites and resulting mature peptide sequences, ii) standardization of mature peptide names, iii) determination of proposed strain genotypes, and iv) detection of potential recombination events. These comprehensive datasets are made freely available for use and download at the ViPR framework at www.viprbrc.org. By providing this data we hope that the rapid and open exchange of ZIKV proteome and genotype information will facilitate and accelerate clinical reagent development.

## Materials and Methods

### Mature peptides

Mature peptide annotation of ZIKV genomes in public databases is currently very sparse, and no tools are available which perform the work required to predict them. However, other flaviviruses such as Dengue have been studied for longer and offer context within the virus family. Therefore we performed sequence similarity analyses between the ZIKV reference genome [4] and genomes of seven closely related species also in the Flavivirus Genus. Specifically, we aligned ZIKV polyprotein with reference polyproteins of Dengue virus, St. Louis encephalitis virus, West Nile virus, Japanese encephalitis virus, Murray Valley encephalitis virus, Usutu virus, and Yellow fever virus (see Table 1 for the sequence accessions). The resulting multiple sequence alignment (MSA) showed very high similarity between the polyprotein of ZIKV and other Flaviviruses, as shown in the Supporting Information (Fig A in S1 File). Importantly, the protease cleavage sites used to generate mature peptides from the seven Flavivirus all align very well (Table A in S1 File). Examination of the large polyprotein MSA showed that the similarity at the P1 position is 100%, either being represented by a basic (K/R) or small (A/S/G) residue, using the cleavage nomenclature convention of Schechter and Berger [5]. Sequence identity across the conserved octapeptide binding site C-terminal to the cut site is highest at the two required basic positions when the viral protease performs the cleavage. The entire P’ substrate leaving group is variable and not rigidly controlling specificity. In total these alignment data imply that the high homology of the protease cut sites can be exploited computationally, to produce reliable results regarding predicted Zika mature peptides.

**Table 1:** ZIKV and other flaviviruses used to identify mature peptides for the ZIKV polyprotein.

Taxon	Viral species	Genome accession	Polyprotein
12637	Dengue virus	NC_002640	NP_073286
11072	Japanese encephalitis virus	NC_001437	NP_059434
11079	Murray Valley encephalitis virus	NC_000943	NP_051124
11080	St. Louis encephalitis virus	NC_007580	YP_001008348
64286	Usutu virus	NC_006551	YP_164264
11082	West Nile virus	NC_001563	NP_041724
11089	Yellow fever virus	NC_002031	NP_041726
64320	Zika virus	NC_012532	YP_002790881

Because the cleavage sites were highly conserved and reliable we developed a Perl script to produce the protease cleavage site coordinates and resultant mature peptide sequences for ZIKV polyproteins. In short, a query polyprotein is aligned with the reference sequence, YP_002790881. The quality of the alignment is then evaluated, and any query with low similarity (<60% conserved amino acid residues) is rejected to mitigate any errant result due to large gaps or insertions in a query virus genome. The start and end positions of the *reference* mature peptides are then used to calculate the start and end positions of the mature peptides in the *query* polyprotein. The amino acid positions are then converted to the nucleotide positions in the genome, and the name of the reference protein product is taken to be the name of the new mature peptide. Any overhang consisting of a partial codon before the start, or after the end of a mature peptide from a partial genome, is adjusted. The amino acid sequences of the resulting mature peptides are stored in the database and provided publicly in the FASTA format through the ViPR workbench facility, so that users can perform further analysis on their own.

### Genotype and recombination

Advances in genome annotation are complementary to an accompanying analysis of virus typing that informs diagnostics, so the present work was extended to include phylogeny studies. ZIKV infections were first reported in Africa, then Southeast Asia, followed by Pacific islands, and most recently in South America [2]. Genomic sequences from different times and geographic locations show distinctions that are clearly seen in a ZIKV phylogenetic tree [6,7, 8]. The branching index of a query sequence in a phylogeny tree [9] has been shown to indicate how closely related such a sequence is to a subtype clade. We adapted this concept to assign genotype calls to available ZIKV genomes. In this analysis, a query genome is aligned with a selected set of reference sequences (Table B in S1 File) to produce a Flavivirus Genus-specific MSA (Fig A in S1 File). The MSA data is then used to computationally infer a phylogenic tree. The location of the query sequence node in the phylogenic tree is used to determine its genotype. Last, to identify any possible recombination event, we defined a sliding window of 400 nucleotides in the alignment, and determine the genotype for each segment. A recombination event is defined when a significant part of the sequence is determined as one genotype, followed by a significant length from another genotype.

### Dataset

Both the mature peptide annotations and the genotype and recombination analyses have been applied to all existing ZIKV genomes in the ViPR database. Annotation to ZIKV genomes made available in the future will also be provided. The dataset and results are freely available through the Zika virus portal (http://www.viprbrc.org/brc/home.spg?decorator=flavi_zika), and can be used in a number of subsequent analyses in the ViPR website (www.viprbrc.org). Moreover, the genotype and recombination analyses can be performed using any user-uploaded sequences.

## Results and Discussion

### ZIKV mature peptide data

**Fig 1:**
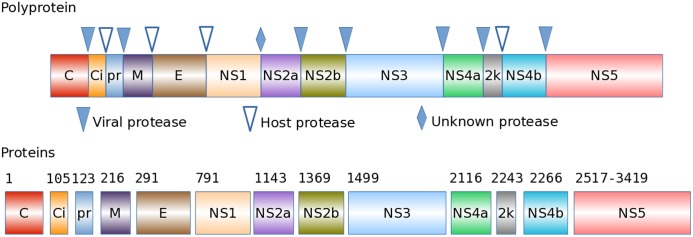
ZIKV polyprotein processing to produce mature peptides. The polyprotein of Zika virus is post-translationally processed by viral and host proteases into functional mature peptides: the capsid protein (C), intracellular capsid protein (Ci), signal peptide of the premembrane protein (pr), membrane protein (M), envelope protein (E), nonstructural proteins (NS1 –NS5), including the proteolytic helicase (NS3) and the RNA-dependent RNA polymerase (NS5), and the 2k protein (2k), whose removal activates the virus particle during its final assembly. The numbers above the mature peptides correspond to the first amino acid of each mature peptide in the full-length polyprotein for genome NC_012532.

ZIKV mature peptides were computed from the parent polyproteins. Like other Flaviviruses, ZIKV encodes a single open reading frame in its single-stranded positive-sense RNA genome (Fig 1). The one continuous open reading frame generates a polyprotein as a primary translation product. After translation, this polyprotein is processed by viral and host proteases into fourteen functional units called mature peptides. It is these mature peptides that control virus biology, including its replication, transmission, pathogenicity, and host immunologic responses, thus they influence disease prognosis. The fourteen ZIKV mature peptides include capsid, membrane, envelope, and nonstructural proteins, as shown in Fig 1. To our knowledge, no mature peptides for ZIKV have been experimentally defined through protein sequencing, or via inference from mass spectrometry. Instead, their presence is derived and substantiated from phylogenetic and comparative analysis, similarities in flavivirus genome synteny, and structure-function analysis of highly similar virus and host proteases. Based on the first complete ZIKV genome [4] and our study of protease cleavage specificities and mature peptide annotation standards in other Flaviviruses [10], we present mature peptide predictions and nomenclature standards for ZIKV below. These new theoretical data can be used to corroborate and predict laboratory results, such as molecular weight and isoelectric point of the resulting peptides, as are also computed at ViPR for these protein products.

Significant support for using this alignment-based strategy exists. The method is guided by the high conservation of Flaviviral cleavage sites, and conservation of the viral NS3 protease itself, and the well known polyprotein cleavage sites from other Flavivirus such as Dengue that could be used as a cutting template, in order to transitively annotate presumptive cuts across the ZIKV genome. Such an effort is performed here *in silico* because of the lack of experimentally validated ZIKV protein sequence data, or proteolytic confirmation that the mature protein amino and carboxy termini are produced as they are in other Flaviviruses. In fact, significant evidence exists that validates a strategy that uses other flaviviral protease cleavage specificities, in concert with their polyprotein substrate conservations. The recent solution of a ZIKV NS3 protease confirmed the conserved catalytic residues H51, D75, and S135, as well as validating our theoretical binding site for two basic residues and a small group (KKGE) in a bound substrate [11]. Analysis of the crystal structure of the ZIKV and West Nile virus NS3 protease show that NS3 hydrolytic specificity is controlled identically by the P4-P1 substrate binding sites. Similarities also exist in Yellow Fever and Murray Valley Fever Virus proteases. Overall, two basic residues precede a cut site in the polyprotein substrate. The preference for a substrate lysine at P2 is due to a salt bridge between that epsilon-amino group and a conserved, negatively charged NS3 Asp83 carboxyl group. A strong preference for a basic substrate residue at P3 is due to another conserved carbonyl oxygen contributed by the NS3 protease Phe84. Enzyme-substrate interactions are nearly identical between WNV and ZIKV [12]. Such sequence conservation occurs at all other NS3 cleavage sites, and even the *least* well understood cut at NS1/NS2 has been well characterized with high confidence in Murray Valley Fever virus [13]. For these reasons we infer that the predicted cuts are valid.

Last, while the theoretical computation of all the mature peptides is straightforward, and should yield valid results, we also put in place a database method to ensure that whenever a known valid mature peptide sequence appears in GenBank, it takes precedence over a theoretically computed sequence. This clearly identifies theoretical and experimentally validated peptides. We ensured that all mature peptides produced are labeled as ViPR-predicted, and the resource clearly labels annotation as such. In fact, this new data should guide experimental validation of predicted mature peptides.

A major outcome of this work is the annotation and presentation of a complete set of ZIKV mature peptide sequences in the publicly accessible Virus Pathogen Resource (ViPR, www.viprbrc.org) database. We developed a pairwise sequence alignment based method that predicts mature peptide products for any complete ZIKV genome. In brief, a query polyprotein is aligned with a reference sequence, the reference cleavage sites are projected onto the strand, and the genomic locations of the mature peptides are calculated. When the protease cleavage sites from other Flaviviruses are projected onto the ZIKV polyprotein in an MSA, the amino acid sequences around the cleavage sites all show highly similar patterns (Table A in S1 File, and Fig A in S1 File) suggesting that protease cleavage is a conserved feature across these Flavivirus species.

Among the twelve cleavage sites, seven are cleaved by the NS3 viral protease: junctions between C/Ci, pr/M, NS2a/NS2b, NS2b/NS3, NS3/NS4a, NS4a/2k, and NS4b/NS5 (Fig 1). Each of these junctions includes two basic amino acids immediately before the cleavage site. The pr/M junction can also potentially be cleaved by host Golgi protease furin [14]. Finally four of the other cleavage sites (junctions between Ci/pr, M/E, E/NS1, and 2k/NS4b) show strong polyprotein sequence conservation across the eight different Flavivirus species. These are presumed to be cleaved by one or more host proteases. NS1/NS2a is cleaved by an unknown protease, but is conserved across the eight different Flavivirus species, and has been well studied in Murray Valley Fever virus [13].

It should be stated that all Zika mature peptide cleavages are not yet supported by experimental sequencing evidence. Almost all the cleavage sites are obvious when inspecting the multiple sequence alignment. However the junction between protein pr and membrane (pr/M) deserves special consideration as it is less well understood, may control pathogenicity, and could possibly be produced by either the host or virus protease. Previous work showed that the host furin protease has looser substrate specificity than the viral NS3 protease, and can cleave at the junction as described here, but also might cut upstream at another basic site [14]. We acknowledge the polyprotein could be cleaved either by the viral or host protease, since at this site the polyprotein retains the basic P3/P2/P1 residues used by both, but also a small C-terminal leaving group residue (A/G/S) at P1' typical of a viral protease cleavage consensus. Rather than computing both possible host cleavage sites, and adding complexity to the data handling, we have chosen the most consistently implied cut based on available sequence conservation and host biology. This cut occurs at the Golgi complex. In one instance in the Murray Valley encephalitis virus, the viral protease is required to be present [15]. However, certain mutations introduced into Tick borne encephalitis virus prM obviate the need for host Golgi protease altogether [16]. Therefore the actual processing of the prM junction remains unknown at this point but does require the viral protease to be present, so for this work we have processed the junction as if the *products were solely from the viral protease*. This approach further makes the data general, and eliminates the complexity of different host species cleaving the junction in slightly different ways. If in the future this approach is overturned and more data presented, then the algorithm will be modified to reflect the cut more accurately.

Using this method, all publicly available genomes have been re-annotated within the ViPR resource. Further, these are made available through a newly developed custom ZIKV portal (http://www.viprbrc.org/brc/home.spg?decorator=flavi_zika). As new ZIKV genomes become available, their mature peptides will be automatically added to the database, so that complete and consistent ZIKV mature peptide sequence data and annotations will always be available to the research community. In May 2016, ViPR contained 1821 ZIKV protein records, more than five times as many as the GenBank Zika resource. By November 2016, ViPR provided over 3300 ZIKV protein records, as compared to 600 in GenBank. Thus, ViPR is providing a new, standardized annotation system for ZIKV mature peptides and the computational methods to generate them.

### Genotype and recombination analysis

**Fig 2:**
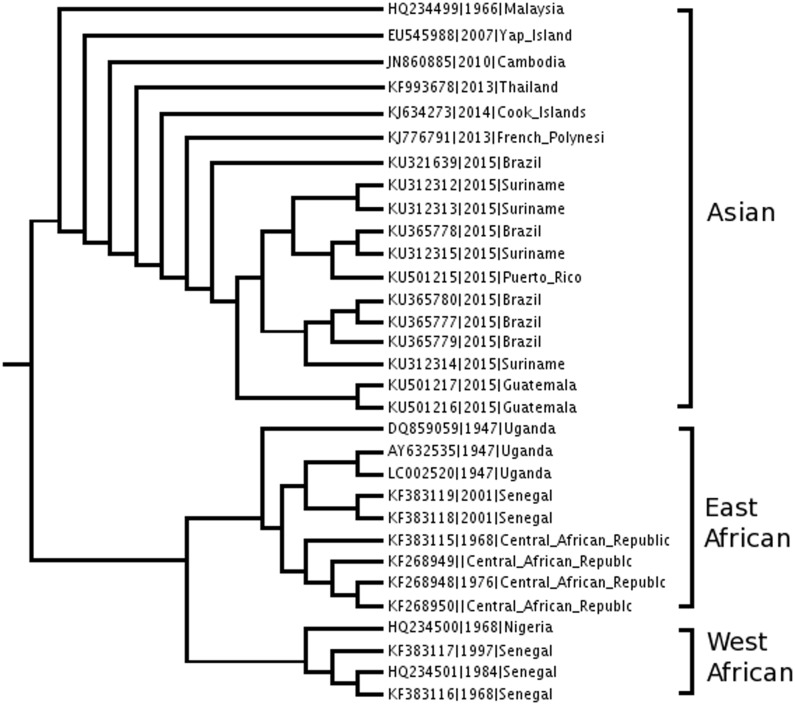
ZIKV reference phylogeny tree and genotypes. Three distinct genotype lineages—Asian, East African and West African—are apparent in the ZIKV phylogeny tree. FastME was used to produce a phylogenetic tree with complete genome nucleotide sequences of a selected set of reference strains. Trees with virtually identical branching structures were produced using RaxML and PhyML (not shown).

The advent of multiple ZIKV genome sequences also makes it possible to analyze their phylogentic relationships. ZIKV strains seem to cluster into distinct genotype lineages [7,8]. Genotype grouping of ZIKV from primary nucleotide sequence will help track transmission chains during the current outbreak and may help differentiate highly pathogenic from less pathogenic virus strains. To facilitate the genotype grouping of ZIKV, a genotyping reference tree was generated based on our phylogenetic analysis of complete ZIKV genomes. Testing of several tree building methods, including RaxML, FastME, PhyML and others resulted in a consensus of three main branches or genotypes: East African, West African, and Asian (Fig 2). To predict the genotype of a query genome, the genomic sequence is combined with the sequences of a selected set of reference sequences to produce a phylogeny tree, and the location of the query genome in the phylogeny tree is used to determine its genotype. All of the ~400 existing ZIKV genomes have been analyzed and the result is available at the ViPR resource. Of the 92 complete ZIKV genomes available as of May 20, 2016, 69 were typed as Asian, 15 as East African, and 8 as West African. The number of Asian genotype genomes submitted to ViPR has more than doubled since then. By November 2016, of the 172 complete genomes in ViPR, 148 were Asian genotypes, but there were an unchanged number of 15 East African and 8 West African genotypes. The data are updated as new genomes are received.

Recombination events have possibly occurred in which at least two different virus lineages contribute to the single-stranded flaviviral RNA genome [17]. Though rare *in vivo*, these events signify co-infection of different viral lineages, and can cause major change in the biology and pathogenicity of a virus, as they have in the 2009 pandemic “triple reassortant” influenza strain. Predictive detection of this event would be advantageous. To develop and integrate such a tool to our resource we have built a bioinformatic method to detect possible recombination events in the ZIKV genomes. The tool works by calculating a genotype profile along the entire length of the sequence, using a 400-nucleotide sliding window approach. Recombination is inferred when a switch in genotype is detected in different genomic regions.

We performed analysis of all 158 currently available full-length ZIKV genomes (as of Nov 2016), and this revealed no complete recombination events. One might expect a single crossover event to produce a major and minor parent, with each genome end coming from one other lineage. While this was not detected, some recombination signal was identified for 3 genomes: KF383117 (in PrM), KF383113 (in PrM), and KF383118 (in Env). The recombination signal represents a possible recombination change due to a segment substitution near the amino terminus of the polyprotein. Other putative recombination events have been described by WHO in a preprint [18], using the RDP4 tool to detect recombination[19]. Notably all these sequences in both analyses were African, not Asian. However in our results the significance of this recombination detection signal is unknown, and represents only 1.9% of all samples. We therefore infer that recombination in Zika virus is a rare event.

## Conclusions

This report describes a suite of bioinformatic tools to predict polyprotein protease cleavage sites, lineage genotypes and recombination events for ZIKV. These tools have been used to produce comprehensive, consistent, and continually updated annotations for all publicly available ZIKV sequences made available through the ViPR resource. These standardized annotations and tools are expected to enhance the rapid dissemination of crucial information needed for the development of diagnostics and treatment interventions for this newly emerging pathogen.

## Supporting Information

S1 File Supporting information for computation of Zika virus mature peptides and genotypes. Detail is provided for the alignment methods, reference sequences, polyprotein proteolytic cleavage sites, as well as the full multiple sequence alignment.
(DOCX)
