# Supplementary material for: Comprehensive Annotation of Mature Peptides and Genotypes for Zika Virus
Source: PLoS One. 2017 Jan 26;12(1):e0170462. doi: 10.1371/journal.pone.0170462 (PMC5268401; doi:10.1371/journal.pone.0170462)
Supplement: S1 File — Detail is provided for the alignment methods, reference sequences, polyprotein proteolytic cleavage sites, as well as the full multiple sequence alignment. (DOCX) [file pone.0170462.s001.docx]

**Supporting Information:**

**Comprehensive Annotation of Mature Peptide and Genotype for Zika Virus**

Guangyu Sun^1^, Christopher N. Larsen^1^, Nicole Baumgarth^2^, Edward B. Klem^3^, and Richard H. Scheuermann^4,5,6^

^1.^ Vecna Technologies, Inc., Greenbelt, Maryland 20770, USA,

^2.^ Center for Comparative Medicine and the Department of Pathology, Microbiology & Immunology, University of California, Davis, Davis, CA 95616, USA,

^3.^ Northrop Grumman Health Solutions, Rockville, Maryland 20850, USA,

^4.^ J. Craig Venter Institute, La Jolla, CA 92037, USA,

^5.^ Department of Pathology, University of California, San Diego, San Diego, CA 92093, USA,

^6^ Division of Vaccine Discovery, La Jolla Institute for Allergy and Immunology, La Jolla, CA 92037, USA

**Multiple sequence alignment of Zika polyprotein with closely related polyproteins**

We used the sequence alignment program MUSCLE [1] to align ZIKV polyprotein with seven polyproteins from closely related species through the web service at [www.viprbrc.org](http://www.viprbrc.org/). The resulting MSA with residue coloring is shown in Fig A. The first five amino acids of a mature peptide are marked in bold only, and the last five amino acids in bold and underlined. Thus the intersection of the bold-underlined segments with the bold segments marks a cleavage site. The seven cleavage sites cleaved by viral protease all follow two basic amino acids before the cleavage. Four other cleavage sites cleaved by host protease show much similarity across the eight polyproteins of different Flaviviruses. The last cleavage site cleaved by unknown protease also shows much similarity across the eight polyproteins.

Additionally, the sequences surround the ZIKV cleavage sites are given in Table A. The sequences around the cleavage sites clearly show the different requirements for viral and host proteases.

**Table A**. **Cleavage sites for ZIKV polyprotein, and their surrounding amino sequences.** The cleavage sites are marked by “|”. Location of the cleavage site in polyprotein YP_002790881 of ZIKV genome NC_012532.

| **ZIKV cleavage site** | **Surround AA sequence^a^** | **Location^b^** | **Protease** |
| --- | --- | --- | --- |
| C/Ci | INARK**ERKRR**\|**GADTS**IGIIG | 104-105 | Viral |
| Ci/pr | IGLLL**TTAMA**\|**AEITR**RGSAY | 122-123 | Host |
| pr/M | KKGEA**RRSRR**\|**AVTLP**SHSTR | 215-216 | Viral or Host |
| M/E | MILLI**APAYS**\|**IRCIG**VSNRD | 290-291 | Host |
| E/NS1 | MIFLS**TAVSA**\|**DVGCS**VDFSK | 790-791 | Host |
| NS1/NS2a | SNLVR**SMVTA**\|**GSTDH**MDHFS | 1142-1143 | Unknown |
| NS2a/NS2b | LLLLT**RSGKR**\|**SWPPS**EVLTA | 1368-1369 | Viral |
| NS2b/NS3 | WYVYV**KTGKR**\|**SGALW**DVPAP | 1498-1499 | Viral |
| NS3/NS4a | SFKEF**AAGKR**\|**GAALG**VMEAL | 2115-2116 | Viral |
| NS4a/2k | VLIPE**PEKQR**\|**SPQDN**QMAII | 2242-2243 | Viral |
| 2k/NS4b | AVGLL**GLITA**\|**NELGW**LERTK | 2265-2266 | Host |
| NS4b/NS5 | TRNAG**LVKRR**\|**GGGTG**ETLGE | 2516-2517 | Viral |

**Fig A**. **Multiple sequence alignment of ZIKV polyprotein with polyproteins from seven closely-related human Flaviviruses**. The cleavage sites are marked with “|”, and the first five amino acids of a mature peptide are in bold, and the last five amino acids are in bold and underlined.

Mature peptide -Capsid

NC_012532|Zika **MKNPK**EEIRRIRIVNMLKRGVARVNPLGGL-KRLPAGLLLGHGPIRMVLAILAFLRFTAI

NC_002640|Dengue **MNQRK**KVVRPP--FNMLKRERNRVSTPQGLVKRFSTGLFSGKGPLRMVLAFITFLRVLSI

NC_001563|West_Nile **MSKKP**GGPGKNRAVNMLKRGMPRGLSLIGL-KRAMLSLIDGKGPIRFVLALLAFFRFTAI

NC_002031|Yellow_fever **MSGRK**AQ-GKTLGVNMVRRGVRSLSNKI---KQKTKQIGNRPGPSRGVQGFIFFFLFNIL

NC_001437|Japanese_enceph **MTKKP**GGPGKNRAINMLKRGLPRVFPLVGV-KRVVMSLLDGRGPVRFVLALITFFKFTAL

NC_006551|Usutu_virus **MSKKP**GGPGRNRAINMLKRGIPRVFPLVGV-KRVVMGLLDGRGPVRFVLALMTFFKFTAL

NC_007580|St_Louis **MSKKP**GKPGRNRVVNMLKRGVSRVNPLTGL-KRILGSLLDGRGPVRFILAILTFFRFTAL

NC_000943|Murray_Valley_enceph **MSKKP**GGPGKPRVVNMLKRGIPRVFPLVGV-KRVVMNLLDGRGPIRFVLALLAFFRFTAL

*. .**:.* *. : ** * : .:: *: . :

Mature peptide Capsid-|-Ci

NC_012532|Zika KP---SLGLINRWGSVGKKEAMEIIKKFKKDLAAMLRIINARK**ER-KRR**|**GADTS**----IG

NC_002640|Dengue PP---TAGILKRWGQLKKNKAIKILIGFRKEIGRMLNIL**NGRKR**|**S-------------TI**

NC_001563|West_Nile AP---TRAVLDRWRGVNKQTAMKHLLSFKKELGTLTSAINRRST**KQKKR**|**GGTAG**----FT

NC_002031|Yellow_fever TGKKITAHLKRLWKMLDPRQGLAVLRKVKRVVASLMRGLS**SRKRR**----|--**SHDVL**TVQF

NC_001437|Japanese_enceph AP---TKALLGRWKAVEKSVAMKHLTSFKRELGTLIDAVNKRGR**KQNKR**|**GGNEG**SIMWLA

NC_006551|Usutu_virus AP---TKALLGRWKRINKTTAMKHLTSFKKELGTMINVVNNRG**TK-KKR**|**GNNGP**GLVMII

NC_007580|St_Louis QP---TEALKRRWRAVDKRTALKHLNGFKRDLGSMLDTINRR**PSK--KR**|**GGTRS**----LL

NC_000943|Murray_Valley_enceph AP---TKALMRRWKSVNKTTAMKHLTSFKKELGTLIDVVNKRGK**KQKKR**|**GGSET**SV--LM

: : * : .: : ... :. : :. * |

Mature peptide Ci-|-precursor Membrane

NC_012532|Zika IIGLLL**TTAMA**|**AEITR**RGSAYYMYLDRSDAGKAISFATTLGVNKCHVQIMDLGHMCDATM

NC_002640|Dengue **TL**LCLI**PTVMA**|**FSLST**RDGEPLMIVAKHERGRPLLFKTTEGINKCTLIAMDLGEMCEDTV

NC_001563|West_Nile ILLGLI**ACAGA**|**VTLSN**FQGKVMMTVNATDVTDVITIPTAAGKNLCIVRAMDVGYLCEDTI

NC_002031|Yellow_fever LILGML**LMTGG**|**VTLVR**KNRWLLLNVTSEDLGKTFSV----GTGNCTTNILEAKYWCPDSM

NC_001437|Japanese_enceph SLAVVI**AYAGA**|**MKLSN**FQGKLLMTINNTDIADVIVIPTSKGENRCWVRAIDVGYMCEDTI

NC_006551|Usutu_virus TLMTVV**SMVSS**|**LKLSN**FQGKVMMTINATDMADVIVVPTQHGKNQCWIRAMDVGYMCDDTI

NC_007580|St_Louis GLAALI**GLASS**|**LQLST**YQGKVLMSINKTDAQSAINIPSANGANTCIVRALDVGVMCKDDI

NC_000943|Murray_Valley_enceph LIFMLI**GFAAA**|**LKLST**FQGKIMMTVNATDIADVIAIPTPKGPNQCWIRAIDIGFMCDDTI

: :: . .| : : : : : . * . * :: * :

Mature peptide precursor Membrane-|-Membrane glycoprotein

NC_012532|Zika SYECPMLDEGVEPDDVDCWC-NTTSTWVVYGTCHHKKGEA**RRSRR**|**AVTLP**SHSTRKLQTR

NC_002640|Dengue TYKCPLL-VNTEPEDIDCWC-NLTSTWVMYGTC-TQSGER**RREKR**|**SVALT**PHSGMGLETR

NC_001563|West_Nile TYECPVLAAGNDPEDIDCWC-TKSSVYVRYGRC-TKTRHS**RRSRR**|**SLTVQ**THGESTLANK

NC_002031|Yellow_fever EYNCPNLSPREEPDDIDCWCYGVENVRVAYGKC-DSAGRS**RRSRR**|**AIDLP**THENHGLKTR

NC_001437|Japanese_enceph TYECPKLTMGNDPEDVDCWC-DNQEVYVQYGRC-TRTRHS**KRSRR**|**SVSVQ**THGESSLVNK

NC_006551|Usutu_virus TYECPKLDAGNDPEDIDCWC-DKQPMYVHYGRC-TRTRHS**KRSRR**|**SIAVQ**THGESMLANK

NC_007580|St_Louis TYLCPVLSAGNDPEDIDCWC-DVEEVWVHYGRC-TRMGHS**RRSRR**|**SISVQ**HHGDSTLATK

NC_000943|Murray_Valley_enceph TYECPKLESGNDPEDIDCWC-DKQAVYVNYGRC-TRARHS**KRSRR**|**SITVQ**THGESTLVNK

* ** * :*:*:**** * ** * .*..*|:: : * * ..

Mature peptide Membrane glycoprotein-|

NC_012532|Zika SQTWLESREYTKHLIKVENWIFRNPGFALVAVAIAWLLGSSTSQKVIYLVMILLI**APAYS**|

NC_002640|Dengue AETWMSSEGAWKHAQRVESWILRNPGFALLAGFMAYMIGQTGIQRTVFFVLMMLV**APSYG**|

NC_001563|West_Nile KGAWLDSTKATRYLVKTESWILRNPGYALVAAVIGWMLGSNTMQRVVFAILLLLV**APAYS**|

NC_002031|Yellow_fever QEKWMTGRMGERQLQKIERWFVRNPFFAVTALTIAYLVGSNMTQRVVIALLVLAV**GPAYS**|

NC_001437|Japanese_enceph KEAWLDSTKATRYLMKTENWIIRNPGYAFLAATLGWMLGSNNGQRVVFTILLLLV**APAYS**|

NC_006551|Usutu_virus KDAWLDSTKASRYLMKTENWIIRNPGYAFVAVLLGWMLGSNNGQRVVFVVLLLLV**APAYS**|

NC_007580|St_Louis NTPWLDTVKTTKYLTKVENWVLRNPGYALVALAIGWMLGSNNTQRVVFVIMLMLI**APAYS**|

NC_000943|Murray_Valley_enceph KDAWLDSTKATRYLTKTENWIIRNPGYALVAVVLGWMLGSNTGQKVIFTVLLLLV**APAYS**|

*: . . * *..*** :*. * :.:::*.. *..: :::: :.*:*.|

Mature peptide -Envelope

NC_012532|Zika **IRCIG**VSNRDFVEGMSGGTWVDVVLEHGGCVTVMAQDKPTVDIELVTTTVSNMAEVRSYC

NC_002640|Dengue **MRCVG**VGNRDFVEGVSGGAWVDLVLEHGGCVTTMAQGKPTLDFELTKTTAKEVALLRTYC

NC_001563|West_Nile **FNCLG**MSNRDFLEGVSGATWVDLVLEGDSCVTIMSKDKPTIDVKMMNMEAANLADVRSYC

NC_002031|Yellow_fever **AHCIG**ITDRDFIEGVHGGTWVSATLEQDKCVTVMAPDKPSLDISLETVAIDRPAEVRKVC

NC_001437|Japanese_enceph **FNCLG**MGNRDFIEGASGATWVDLVLEGDSCLTIMANDKPTLDVRMINIEASQLAEVRSYC

NC_006551|Usutu_virus **FNCLG**MSNRDFLEGVSGATWVDVVLEGDSCITIMAKDKPTIDIKMMETEATNLAEVRSYC

NC_007580|St_Louis **FNCLG**TSNRDFVEGASGATWIDLVLEGGSCVTVMAPEKPTLDFKVMKMEATELATVREYC

NC_000943|Murray_Valley_enceph **FNCLG**MSSRDFIEGASGATWVDLVLEGDSCITIMAADKPTLDIRMMNIEATNLALVRNYC

.*:* .***:** *.:*:. .** . *:* *: **::*. : * :* *

NC_012532|Zika YEASISDMASDSRCPTQGEAYLDKQSDTQYVCKRTLVDRGWGNGCGLFGKGSLVTCAKFT

NC_002640|Dengue IEASISNITTATRCPTQGEPYLKEEQDQQYICRRDVVDRGWGNGCGLFGKGGVVTCAKFS

NC_001563|West_Nile YLASVSDLSTRAACPTMGEAHNEKRADPAFVCKQGVVDRGWGNGCGLFGKGSIDTCAKFA

NC_002031|Yellow_fever YNAVLTHVKINDKCPSTGEAHLAEENEGDNACKRTYSDRGWGNGCGLFGKGSIVACAKFT

NC_001437|Japanese_enceph YHASVTDISTVARCPTTGEAHNEKRADSSYVCKQGFTDRGWGNGCGLFGKGSIDTCAKFS

NC_006551|Usutu_virus YLATVSDVSTVSNCPTTGEAHNPKRAEDTYVCKSGVTDRGWGNGCGLFGKGSIDTCANFT

NC_007580|St_Louis YEATLDTLSTVARCPTTGEAHNTKRSDPTFVCKRDVVDRGWGNGCGLFGKGSIDTCAKFT

NC_000943|Murray_Valley_enceph YAATVSDVSTVSNCPTTGESHNTKRADHNYLCKRGVTDRGWGNGCGLFGKGSIDTCAKFT

* : : **: **.: : : *. **************.: :**:*:

NC_012532|Zika CSKKMTGKSIQPENLEYRIMLSVHGSQHSGMIGYETD----EDRAKVEVTPNSPRAEATL

NC_002640|Dengue CSGKITGNLVQIENLEYTVVVTVHNG-DTHAVGNDTS----NHGVTAMITPRSPSVEVKL

NC_001563|West_Nile CTTKATGWIIQKENIKYEVAIFVHGPTTVESHG----KIGATQAGRFSITPSAPSYTLKL

NC_002031|Yellow_fever CAKSMSLFEVDQTKIQYVIRAQLHVGAKQENWN--------TDIKTLKFDALSGSQEVEF

NC_001437|Japanese_enceph CTSKAIGRTIQPENIKYEVGIFVHGTTTSENHGNYSAQVGASQAAKFTITPNAPSITLKL

NC_006551|Usutu_virus CSLKAMGRMIQPENVKYEVGIFIHGSTSSDTHGNYSSQLGASQAGRFTITPNSPAITVKM

NC_007580|St_Louis CKNKATGKTILRENIKYEVAIFVHGSTDSTSHGNYSEQIGKNQAARFTISPQAPSFTANM

NC_000943|Murray_Valley_enceph CSNSAAGRLILPEDIKYEVGVFVHGSTDSTSHGNYSTQIGANQAVRFTISPNAPAITAKM

* . : .::* : :* . . . : :

NC_012532|Zika GGFGSLGLDCEPRTGLDFSDLYYLTMNNKHWLVHKEWFHDIPLPWHAGADTGTPHWNNKE

NC_002640|Dengue PDYGELTLDCEPRSGIDFNEMILMKMKKKTWLVHKQWFLDLPLPWTAGADTSEVHWNYKE

NC_001563|West_Nile GEYGEVTVDCEPRSGIDTSAYYVMSVGEKSFLVHREWFMDLNLPW---SSAGSTTWRNRE

NC_002031|Yellow_fever IGYGKATLECQVQTAVDFGNSYIAEMETESWIVDRQWAQDLTLPW---QSGSGGVWREMH

NC_001437|Japanese_enceph GDYGEVTLDCEPRSGLNTEAFYVMTVGSKSFLVHREWFHDLALPW---TSPSSTAWRNRE

NC_006551|Usutu_virus GDYGEISVECEPRNGLNTEAYYIMSVGTKHFLVHREWFNDLALPW---TSPASSNWRNRE

NC_007580|St_Louis GEYGTVTIDCEARSGINTEDYYVFTVKEKSWLVNRDWFHDLNLPW---TSPATTDWRNRE

NC_000943|Murray_Valley_enceph GDYGEVTVECEPRSGLNTEAYYVMTIGTKHFLVHREWFNDLLLPW---TSPASTEWRNRE

:* ::*: ...:: : : ::* .:* *: *** . . *.

NC_012532|Zika ALVEFKDAHAKRQTVVVLGSQEGAVHTALAGALEAEMDGAKG---RLFSGHLKCRLKMDK

NC_002640|Dengue RMVTFKVPHAKRQDVTVLGSQEGAMHSALAGATEVD-SGDGN---HMFAGHLKCKVRMEK

NC_001563|West_Nile TLMEFEEPHATKQSVVALGSQEGALHQALAGAIPVEFSSNTV---KLTSGHLKCRVKMEK

NC_002031|Yellow_fever HLVEFEPPHAATIRVLALGNQEGSLKTALTGAMRVTKDTNDNNLYKLHGGHVSCRVKLSA

NC_001437|Japanese_enceph LLMEFEEAHATKQSVVALGSQEGGLHQALAGAIVVEYSSS-V---KLTSGHLKCRLKMDK

NC_006551|Usutu_virus ILLEFEEPHATKQSVVALGSQEGALHQALAGAVPVSFSGS-V---KLTSGHLKCRVKMEK

NC_007580|St_Louis TLVEFEEPHATKQTVVALGSQEGALHTALAGAIPATVSSSTL---TLQSGHLKCRAKLDK

NC_000943|Murray_Valley_enceph ILVEFEEPHATKQSVVALGSQEGALHQALAGAIPVEFSSSTL---KLTSGHLKCRVKMEK

:: *: .** * .**.***.:: **:** . . : .**:.*. .:.

NC_012532|Zika LRLKGVSYSLCTAAFTFTKVPAETLHGTVTVEVQYAGTDGPCKIPVQMAVDMQTLTPVGR

NC_002640|Dengue LRIKGMSYTMCSGKFSIDKEMAETQHGTTVVKVKYEGAGAPCKVPIEI-RDVNKEKVVGR

NC_001563|West_Nile LQLKGTTYGVCSKAFKFARTPADTGHGTVVLELQYTGTDGPCKVPISSVASLNDLTPVGR

NC_002031|Yellow_fever LTLKGTSYKICTDKMFFVKNPTDTGHGTVVMQVKVS-KGAPCRIPVIVADDLTAAINKGI

NC_001437|Japanese_enceph LALKGTTYGMCTEKFSFAKNPADTGHGTVVIELSYSGSDGPCKIPIVSVASLNDMTPVGR

NC_006551|Usutu_virus LTLKGTTYGMCTEKFSFAKNPADTGHGTVVLELQYTGSDGPCKIPISIVASLSDLTPIGR

NC_007580|St_Louis VKIKGTTYGMCDSAFTFSKNPTDTGHGTVIVELQYTGSNGPCRVPISVTANLMDLTPVGR

NC_000943|Murray_Valley_enceph LKLKGTTYGMCTEKFTFSKNPADTGHGTVVLELQYTGSDGPCKIPISSVASLNDMTPVGR

: :** :* :* : : . ::* ***. :::. ..**.:*: .: *

NC_012532|Zika LITANPVITESTENSKMMLELDPPFGDSYIVIGVGDKKITHHWHRSGSTIGKAFEATVRG

NC_002640|Dengue IISSTPL--AENTNSVTNIELEPPFGDSYIVIGVGNSALTLHWFRKGSSIGKMFESTYRG

NC_001563|West_Nile LVTVNPFVSVATANSKVLIELEPPFGDSYIVVGRGEQQINHHWHKSGSSIGKAFTTTLRG

NC_002031|Yellow_fever LVTVNPI--ASTNDDEVLIEVNPPFGDSYIIVGRGDSRLTYQWHKEGSSIGKLFTQTMKG

NC_001437|Japanese_enceph LVTVNPFVATSSANSKVLVEMEPPFGDSYIVVGRGDKQINHHWHKAGSTLGKAFSTTLKG

NC_006551|Usutu_virus MVTANPYVASSEANAKVLVEMEPPFGDSYIVVGRGDKQINHHWHKAGSSIGKAFITTIKG

NC_007580|St_Louis LVTVNPFISTGGANNKVMIEVEPPFGDSYIVVGRGTTQINYHWHKEGSSIGKALATTWKG

NC_000943|Murray_Valley_enceph MVTANPYVASSTANAKVLVEIEPPFGDSYIVVGRGDKQINHHWHKEGSSIGKAFSTTLKG

::: .* : :*::********::* * :. :*.. **::** : * .*

NC_012532|Zika AKRMAVLGDTAWDFGSVGGVFNSLGKGIHQIFGAAFKSLFGGMSWFSQILIGTLLVWLGL

NC_002640|Dengue AKRMAILGETAWDFGSVGGLFTSLGKAVHQVFGSVYTTMFGGVSWMIRILIGFLVLWIGT

NC_001563|West_Nile AQRLAALGDTAWDFGSVGGVFTSVGKAIHQVFGGAFRSLFGGMSWITQGLLGALLLWMGI

NC_002031|Yellow_fever VERLAVMGDTAWDFSSAGGFFTSVGKGIHTVFGSAFQGLFGGLNWITKVIMGAVLIWVGI

NC_001437|Japanese_enceph AQRLAALGDTAWDFGSIGGVFNSIGKAVHQVFGGAFRTLFGGMSWITQGLMGALLLWMGV

NC_006551|Usutu_virus AQRLAALGDTAWDFGSVGGIFNSVGKAVHQVFGGAFRTLFGGMSWITQGLMGALLLWMGV

NC_007580|St_Louis AQRLAVLGDTAWDFGSIGGVFNSIGKAVHQVFGGAFRTLFGGMSWITQGLLGALLLWMGL

NC_000943|Murray_Valley_enceph AQRLAALGDTAWDFGSVGGVFNSIGKAVHQVFGGAFRTLFGGMSWISPGLLGALLLWMGV

.:*:* :*:*****.* **.*.*:**.:* :**..: :***:.*: ::* :::*:*

Mature peptide Envelope-|-NS1

NC_012532|Zika NTKNGSISLTCLALGGVMIFLS**TAVSA**|**DVGCS**VDFSKKETRCGTGVFIYNDVEAWRDRYK

NC_002640|Dengue NSRNTSMAMTCIAVGGITLFLG**FTVQA**|**DMGCV**ASWSGKELKCGSGIFVVDNVHTWTEQYK

NC_001563|West_Nile NARDRSIAMTFLAVGGVLLFLS**VNVHA**|**DTGCA**IDIGRQELRCGSGVFIHNDVEAWMDRYK

NC_002031|Yellow_fever NTRNMTMSMSMILVGVIMMFLS**LGVGA**|**DQGCA**INFGKRELKCGDGIFIFRDSDDWLNKYS

NC_001437|Japanese_enceph NARDRSIALAFLATGGVLVFLA**TNVHA**|**DTGCA**IDITRKEMRCGSGIFVHNDVEAWVDRYK

NC_006551|Usutu_virus NARDRSIALVMLATGGVLLFLA**TNVHA**|**DSGCA**IDVGRRELRCGQGIFIHNDVEAWVDRYK

NC_007580|St_Louis QARDRSISLTLLAVGGILIFLA**TSVQA**|**DSGCA**IDLQRRELKCGGGIFVYNDVEKWKSDYK

NC_000943|Murray_Valley_enceph NARDKSIALAFLATGGVLLFLA**TNVHA**|**DTGCA**IDITRRELKCGSGIFIHNDVEAWIDRYK

::.: :::: : * : :**. * *|* ** . .* .** *:*: : * . *.

NC_012532|Zika YHPDSPRRLAAAVKQAWEEGICGISSVSRMENIMWKSVEGELNAILEENGVQLTVVVGSV

NC_002640|Dengue FQPESPARLASAILNAHKDGVCGIRSTTRLENVMWKQITNELNYVLWEGGHDLTVVAGDV

NC_001563|West_Nile FYPETPQGLAKIIQKAHAEGVCGLRSVSRLEHQMWEAIKDELNTLLKENGVDLSVVVEKQ

NC_002031|Yellow_fever YYPEDPVKLASIVKASFEEGKCGLNSVDSLEHEMWRSRADEINAIFEENEVDISVVVQDP

NC_001437|Japanese_enceph YLPETPRSLAKIVHKAHKEGVCGVRSVTRLEHQMWEAVRDELNVLLKENAVDLSVVVNKP

NC_006551|Usutu_virus FMPETPKQLAKVIEQAHAKGICGLRSVSRLEHVMWENIRDELNTLLRENAVDLSVVVEKP

NC_007580|St_Louis YFPLTPTGLARVIQEAHANGICGIRSTSRLEHLMWENIQRELNAIFEDNEIDLSVVVQED

NC_000943|Murray_Valley_enceph YLPETPKQLAKVVENAHKSGICGIRSVNRFEHQMWESVRDELNALLKENAIDLSVVVEKQ

: * * ** : : .* **: *. :*: ** *:* :: :. :::**. .

NC_012532|Zika KNPMWRGPQRLPVPVNELPHGWKAWGKSYFVRAAKTNNSFVVDGDTLKECPLEHRAWNSF

NC_002640|Dengue KGVLTKGKRALTPPVSDLKYSWKTWGKAKIFTPEARNSTFLIDGPDTSECPNERRAWNSL

NC_001563|West_Nile NGMYKAAPKRLAATTEKLEMGWKAWGKSIIFAPELANNTFVIDGPETEECPTANRAWNSM

NC_002031|Yellow_fever KNVYQRGTHPFSRIRDGLQYGWKTWGKNLVFSPGRKNGSFIIDGKSRKECPFSNRVWNSF

NC_001437|Japanese_enceph VGRYRSAPKRLSMTQEKFEMGWKAWGKSILFAPELANSTFVVDGPETKECPDEHRAWNSM

NC_006551|Usutu_virus KGMYKSAPQRLALTSEEFEIGWKAWGKSLVFAPELANHTFVVDGPETKECPDAKRAWNSL

NC_007580|St_Louis PKYYKRAPRRLKKLEDELDYGWKKWGKTLFVEPRLGNNTFVVDGPETKECPTANRAWNSF

NC_000943|Murray_Valley_enceph KGMYRAAPNRLRLTVEELDIGWKAWGKSLLFAAELANSTFVVDGPETAECPNSKRAWNSF

. . : . : .** *** .. . * :*::** *** .*.***:

NC_012532|Zika LVEDHGFGVFHTSVWLKVREDYSLECDPAVIGTAVKGREAAHSDLGYWIESEK-NDTWRL

NC_002640|Dengue EVEDYGFGMFTTNIWMKFREGSSEVCDHRLMSAAIKDQKAVHADMGYWIESSK-NQTWQI

NC_001563|West_Nile EVEDFGFGLTSTRMFLRIRETNTTECDSKIIGTAVKNNMAVHSDLSYWIESGL-NDTWKL

NC_002031|Yellow_fever QIEEFGTGVFTTRVYMDAVFEYTIDCDGSILGAAVNGKKSAHGSPTFWMGSHEVNGTWMI

NC_001437|Japanese_enceph QIEDFGFGITSTRVWLKIREESTDECDGAIIGTAVKGHVAVHSDLSYWIESRY-NDTWKL

NC_006551|Usutu_virus EIEDFGFGIMSTRVWLKVREHNTTDCDSSIIGTAVKGDIAVHSDLSYWIESHK-NTTWRL

NC_007580|St_Louis KVEDFGFGMVFTRLWLTIREENTTECDSAIIGTAIKGDRAVHSDLSYWIESKK-NETWQL

NC_000943|Murray_Valley_enceph EIEDFGFGITSTRGWLKLREENTSECDSTIIGTAVKGNHAVHSDLSYWIESGL-NGTWKL

:*:.* *: * :: : ** ::.:*::. :.*.. :*: * * ** :

NC_012532|Zika KRAHLIEMKTCEWPKSHTLWTDGVEESDLIIPKSLAGPLSHHNTREGYRTQVKGPWHSEE

NC_002640|Dengue EKASLIEVKTCLWPKTHTLWSNGVLESQMLIPKSYAGPFSQHNYRQGYATQTVGPWHLGK

NC_001563|West_Nile ERAVLGEVKSCTWPETHTLWGDGVLESDLIIPITLAGPRSNHNRRPGYKTQNQGPWDEGR

NC_002031|Yellow_fever HTLEALDYKECEWPLTHTI-GTSVEESEMFMPRSIGGPVSSHNHIPGYKVQTNGPWMQVP

NC_001437|Japanese_enceph ERAVFGEVKSCTWPETHTLWGDGVEESELIIPHTIAGPKSKHNRREGYKTQNQGPWDENG

NC_006551|Usutu_virus ERAVFGEIKSCTWPETHTLWSDGVVESDLVVPVTLAGPKSNHNRREGYKVQSQGPWDEED

NC_007580|St_Louis ERAVMGEVKSCTWPETHTLWGDGVVESEMIIPVTLGGPKSHHNKRNGYYTQTKGPWSEGE

NC_000943|Murray_Valley_enceph ERAIFGEVKSCTWPETHTLWGDAVEETELIIPVTLAGPRSKHNRREGYKVQVQGPWDEED

: * * ** :**: .* *:::.:* : .** * ** ** .* ***

NC_012532|Zika LEIRFEECPGTKVYVEETCGTRGPSLRSTTASGRVIEEWCCRECTMPPLSFRAKDGCWYG

NC_002640|Dengue LEIDFGECPGTTVTIQEDCDHRGPSLRTTTASGKLVTQWCCRSCTMPPLRFLGEDGCWYG

NC_001563|West_Nile VEIDFDYCPGTTVTISDSCEHRGPAARTTTESGKLITDWCCRSCTLPPLRFQTENGCWYG

NC_002031|Yellow_fever LEVKREACPGTSVIIDGNCDGRGKSTRSTTDSGKVIPEWCCRSCTMPPVSFHGSDGCWYP

NC_001437|Japanese_enceph IVLDFDYCPGTKVTITEDCGKRGPSVRTTTDSGKLITDWCCRSCSLPPLRFRTENGCWYG

NC_006551|Usutu_virus IVLDFDYCPGTTVTITEACGKRGPSIRTTTSSGRLVTDWCCRSCTLPPLRYRTKNGCWYG

NC_007580|St_Louis IILDFDYCPGTTVTVTEHCGNRGASLRTTTASGKLVTDWCCRSCSLPPLRYTTKDGCWYG

NC_000943|Murray_Valley_enceph IKLDFDYCPGTTVTVSEHCGKRGPSVRTTTDSGKLVTDWCCRSCTLPPLRFTTASGCWYG

: : ****.* : * ** : *:** **.:: :****.*::**: : .****

Mature peptide NS1-|-NS2a

NC_012532|Zika MEIRPRKEPESNLVR**SMVTA**|**GSTDH**MDHFSLGVLVILLMVQEGLKKRMTTKIIMSTSMAV

NC_002640|Dengue MEIRPLSEKEENMVK**SQVTA**|**GQGT-S**ETFSMGLLCLTLFVEECLRRRVTRKHMILVVVIT

NC_001563|West_Nile MEIRPTRHDEKTLVQ**SRVNA**|**YNADM**IDPFQLGLMVVFLATQEVLRKRWTAKISIPAIMLA

NC_002031|Yellow_fever MEIRPRKTHESHLVR**SWVTA**|**GE---IHA**VPFGLVSMMIAMEVVLRKRQGPKQMLVGGVVL

NC_001437|Japanese_enceph MEIRPVRHDETTLVR**SQVDA**|**FNGEM**VDPFQLGLLVMFLATQEVLRKRWTARLTIPAVLGA

NC_006551|Usutu_virus MEIRPMKHDETTLVK**SSVSA**|**HRSDM**IDPFQLGLLVMFLATQEVLRKRWTARLTVPAIVGA

NC_007580|St_Louis MEIRPVKEEEAKLVK**SRVTA**|**GVAGG**MEPFQLGLLVAFIATQEVLKRRWTGKLTLTSLAVC

NC_000943|Murray_Valley_enceph MEIRPMKHDESTLVK**SRVQA**|**FNGDM**IDPFQLGLLVMFLATQEVLRKRWTARLTLPAAVGA

***** * :*.* * *| . :*:: : : *..* . :

NC_012532|Zika LVVMILGGFSMSDLAKLVILMGATFAEMNTGGDVAHLALVAAFKVRPALLVSFIFRANWT

NC_002640|Dengue LCAIILGGLTWMDLLRALIMLGDTMSG-RIGGQ-IHLAIMAVFKMSPGYVLGVFLR-KLT

NC_001563|West_Nile LLVLVFGGITYTDVLRYVILVGAAFAEANSGGDVVHLALMATFKIQPVFLVASFLKARWT

NC_002031|Yellow_fever LGAMLVGQVTLLDLLKLTVAVGLHFHEMNNGGDAMYMALIAAFSIRPGLLIGFGLRTLWS

NC_001437|Japanese_enceph LLVLMLGGITYTDLARYVVLVAAAFAEANSGGDVLHLALIAVFKIQPAFLVMNMLSTRWT

NC_006551|Usutu_virus LLVLILGGITYTDLLRYVLLVGAAFAEANSGGDVVHLALIAAFKIQPGFLAMTFLRGKWT

NC_007580|St_Louis LALLIFGNLTYMDLVRYLVLVGTAFAEMNTGGDVIHLALVAVFKVQPAFLAGLFLRMQWS

NC_000943|Murray_Valley_enceph LLVLLLGGITYTDLVRYLILVGSAFAESNNGGDVIHLALIAVFKVQPAFLVASLTRSRWT

* ::.* .: *: . : :. : . **: ::*::*.*.: * : :

NC_012532|Zika PRESMLLALASCLLQTAISA----LEGDLMVLINGFALAWLAIRAMAVPRTDNIALPILA

NC_002640|Dengue SRETALMVIGMAMTTVLS------IPHDLMELIDGISLGLILLKIVTQFDNTQVGTLALS

NC_001563|West_Nile NQESILLMLAAAFFQMAYYDAKNVLSWEVPDVLNSLSVAWMILRAISFTNTSNVVVPLLA

NC_002031|Yellow_fever PRERLVLTLGAAMVEIALGG----VMGGLWKYLNAVSLCILTINAVASRKASNTILPLMA

NC_001437|Japanese_enceph NQENVVLVLGAALFQLASVD----LQIGVHGILNAAAIAWMIVRAITFPTTSSVTMPVLA

NC_006551|Usutu_virus NQENILLALGAAFFQMAATD----LNFSLPGILNATATAWMLLRAATQPSTSAIVMPLLC

NC_007580|St_Louis NQENILMVIGAAFLQMAAND----LKLEVLPILNAMSIAWMLIRAMKEGKVAMYALPILC

NC_000943|Murray_Valley_enceph NQENLVLVLGAAFFQMAASD----LELTIPGLLNSAATAWMVLRAMAFPSTSAIAMPMLA

.* :: :. .: : : ::. : : :. :.

NC_012532|Zika ALTP--LARGTLLVAWRAGLATCGGIMLLSLKGKGSVKKNLPFVMALGLTAVRVVDPINV

NC_002640|Dengue LTFI--RSTMPLVMAWRTIMAVLFVVTLIPLCRTSCLQKQSHWVEITALILGAQALPVYL

NC_001563|West_Nile LLTP--GLKCLNLDVYRILLLMVGVGSLIKEKRSSAAKKKGACLICLALASTGVFNPMIL

NC_002031|Yellow_fever LLTPVTMAEVRLAAMFFCAVVIIGV--LHQNFKDTSMQKTIPLVALTLTSYLGLTQPFLG

NC_001437|Japanese_enceph LLTP--GMRALYLDTYRIILLVIGICSLLQERKKTMAKKKGAVLLGLALTSTGWFSPTTI

NC_006551|Usutu_virus LLAP--GMRLLYLDTYRITLIIIGICSLIGERRRAAAKKKGAVLLGLALTSTGQFSASVM

NC_007580|St_Louis ALTP--GMRMAGLDVIRCLLLIIGIVTLLNERRESVAKKKGGYLLAAALCQAGVCSPLIM

NC_000943|Murray_Valley_enceph MLAP--GMRMLHLDTYRIVLLLIGICSLLNERRRSVEKKKGAVLIGLALTSTGYFSPTIM

: * :* : .

Mature peptide NS2a-|-NS2b

NC_012532|Zika V-GLLLLT**RSGKR**|**SWPPS**EVLTAVGLICALAGGFAKADIE-MAGPMAAVGLLIVSYVVSG

NC_002640|Dengue ---MTLMK**GASRR**|**SWPLN**EGIMAVGLVSLLGSALLKNDVP-LAGPMVAGGLLLAAYVMSG

NC_001563|West_Nile AAGLMACD**PNRKR**|**GWPAT**EVMTAVGLMFAIVGGLAELDIDSMAIPMTIAGLMFAAFVISG

NC_002031|Yellow_fever LCAFLATR**IFGRR**|**SIPVN**EALAAAGLVGVLA-GLAFQEMENFLGPIAVGGLLMMLVSVAG

NC_001437|Japanese_enceph AAGLMVCN**PNKKR**|**GWPAT**EFLSAVGLMFAIVGGLAELDIESMSIPFMLAGLMAVSYVVSG

NC_006551|Usutu_virus AAGLMACN**PNKKR**|**GWPAT**EVLTAVGLMFAIVGGLAELDVDSMSIPFVLAGLMAVSYTISG

NC_007580|St_Louis MGGLILAH**PNGKR**|**SWPAS**EVLTGVGLMCALAGGLLEFEETSMVVPFAIAGLMYITYTVSG

NC_000943|Murray_Valley_enceph AAGLMICN**PNKKR**|**GWPAT**EVLTAVGLMFAIVGGLAELDIDSMSVPFTIAGLMLVSYVISG

: .*|. * .* : ..**: : .: : : *: **: ::*

NC_012532|Zika KSVDMYIERAGDITWEKDAEVTGNSPRLDVALDESGDFSLVEEDGPPMREIILKVVLMAI

NC_002640|Dengue SSADLSLEKAANVQWDEMADITGSSPIVEVKQDEDGSFSIRDVEETNMITLLVKLALITV

NC_001563|West_Nile KSTDMWIERTADITWESDAEITGSSERVDVRLDDDGNFQLMNDPGAPWKIWMLRMACLAI

NC_002031|Yellow_fever RVDGLELKKLGEVSWEEEAEISGSSARYDVALSEQGEFKLLSEEKVPWDQVVMTSLALVG

NC_001437|Japanese_enceph KATDMWLERAADISWEMDAAITGSSRRLDVKLDDDGDFHLIDDPGVPWKVWVLRMSCIGL

NC_006551|Usutu_virus KSTDLWLERAADITWETDAAITGTSQRLDVKLDDDGDFHLINDPGVPWKIWVIRMTALGF

NC_007580|St_Louis KAAEMWIEKAADITWEQNAEITGTSPRLDVDLDSHGNFKLLNDPGAPVHLFALRFILLGL

NC_000943|Murray_Valley_enceph KATDMWLERAADVSWEAGAAITGTSERLDVQLDDDGDFHLLNDPGVPWKIWVLRMTCLSV

: ::. .:: *: * ::*.* :* .. *.* : . : :

Mature peptide NS2b-|-NS3

NC_012532|Zika CGMNPIAIPFAAGAWYVYV**KTGKR**|**SG-ALW**DVPAPKEVKKGE-TTDGVYRVMTRRLLGST

NC_002640|Dengue SGLYPLAIPVTMTLWYMWQ**VKTQR**|**SG-ALW**DVPSPAATKKAA-LSEGVYRIMQRGLFGKT

NC_001563|West_Nile SAYTPWAILPSVIGFWITL**QYTKR**|**GG-VLW**DTPSPKEYKKGD-TTTGVYRIMTRGLLGSY

NC_002031|Yellow_fever AALHPFALLLVLAGWLFHV**RGARR**|**SGDVL**WDIPTPKIIEECEHLEDGIYGIFQSTFLGAS

NC_001437|Japanese_enceph AALTPWAIVPAAFGYWLTL**KTTKR**|**GG-VFW**DTPSPKPCSKGD-TTTGVYRIMARGILGTY

NC_006551|Usutu_virus AAWTPWAIIPAGIGYWLTV**KYAKR**|**GG-VFW**DTPAPRTYPKGD-TSPGVYRIMSRYILGTY

NC_007580|St_Louis SARFHWFIPFGVLGFWLLG**KHSKR**|**GG-ALW**DVPSPKVYPKCE-TKPGIYRIMTRGILGTF

NC_000943|Murray_Valley_enceph AAITPRAILPSAFGYWLTL**KYTKR**|**GG-VFW**DTPSPKVYPKGD-TTPGVYRIMARGILGRY

.. : : . .*|.* .:** *:* : *:* :: ::*

NC_012532|Zika QVGVGVMQEGVFHTMWHVTKGAALRSGEGRLDPYWGDVKQDLVSYCGPWKLDAAWDGLSE

NC_002640|Dengue QVGVGIHMEGVFHTMWHVTRGSVICHETGRLEPSWADVRNDMISYGGGWRLGDKWDKEED

NC_001563|West_Nile QAGAGVMVEGVFHTLWHTTKGAALMSGEGRLDPYWGSVKEDRLCYGGPWKLQHKWNGHDE

NC_002031|Yellow_fever QRGVGVAQGGVFHTMWHVTRGAFLVRNGKKLIPSWASVKEDLVAYGGSWKLEGRWDGEEE

NC_001437|Japanese_enceph QAGVGVMYENVFHTLWHTTRGAAIMSGEGKLTPYWGSVKEDRIAYGGPWRFDRKWNGTDD

NC_006551|Usutu_virus QAGVGVMYEGVLHTLWHTTRGAAIRSGEGRLTPYWGSVKEDRITYGGPWKFDRKWNGLDD

NC_007580|St_Louis QAGVGVMHEGVFHTMWHATEGAVLRNGEGRLDPYAGDVRNDLISYGGPWKLSATWDGTEE

NC_000943|Murray_Valley_enceph QAGVGVMHEGVFHTLWHTTRGAAIMSGEGRLTPYWGNVKEDRVTYGGPWKLDQKWNGVDD

* *.*: .*:**:**.* *: : .* * ..*.:* : * * *.: *: .:

NC_012532|Zika VQLLAVPPGERARNIQTLPGIFKTKD-GDIGAVALDYPAGTSGSPILDKCGRVIGLYGNG

NC_002640|Dengue VQVLAIEPGKNPKHVQTKPGLFKTLT-GEIGAVTLDFKPGTSGSPIINRKGKVIGLYGNG

NC_001563|West_Nile VQMIVVEPGKNVKNVQTKPGVFKTPE-GEIGAVTLDYPTGTSGSPIVDKNGDVIGLYGNG

NC_002031|Yellow_fever VQLIAAVPGKNVVNVQTKPSLFKVRNGGEIGAVALDYPSGTSGSPIVNRNGEVIGLYGNG

NC_001437|Japanese_enceph VQVIVVEPGKAAVNIQTKPGVFRTPF-GEVGAVSLDYPRGTSGSPILDSNGDIIGLYGNG

NC_006551|Usutu_virus VQLIIVAPGKAAINIQTKPGIFKTPQ-GEIGAVSLDYPEGTSGSPILDKNGDIVGLYGNG

NC_007580|St_Louis VQMIAVAPGKPAINVQTTPGVFKTPL-GTIGAVTLDFPKGTSGSPIINKKGEIIGLYGNG

NC_000943|Murray_Valley_enceph VQMIVVEPGKPAINVQTKPGIFKTAH-GEIGAVSLDYPIGTSGSPIVNSNGEIIGLYGNG

**:: **: ::** *.:*.. * :***:**: *******:: * ::******

NC_012532|Zika VVIKNGSYVSAITQGKREEETPVECFE-PSMLKKKQLTVLDLHPGAGKTRRVLPEIVREA

NC_002640|Dengue VVTKSGDYVSAITQAERIGEPDYEVDE--DIFRKKRLTIMDLHPGAGKTKRILPSIVREA

NC_001563|West_Nile VIMPNGSYISAIVQGERMEEPAPAGFE-PEMLRKKQITVLDLHPGAGKTRKILPQIIKEA

NC_002031|Yellow_fever ILVGDNSFVSAISQTEVKEEGKEELQEIPTMLKKGMTTVLDFHPGAGKTRRFLPQILAEC

NC_001437|Japanese_enceph VELGDGSYVSAIVQGDRQEEPVPEAYT-PNMLRKRQMTVLDLHPGSGKTRKILPQIIKDA

NC_006551|Usutu_virus VILGNGSYVSAIVQGEREEEPVPEAYN-ADMLRKKQLTVLDLHPGAGKTRRILPQIIKDA

NC_007580|St_Louis VLIGQGEYVSGIIQGERTEEPIPDAYN-EEMLRKRKLTVLELHPGAGKTRKVLPQIIKDC

NC_000943|Murray_Valley_enceph VILGNGAYVSAIVQGERVEEPVPEAYN-PEMLKKRQLTVLDLHPGAGKTRRILPQIIKDA

: .. ::*.* * . * ::.* *::::***:***...**.*: :.

NC_012532|Zika IKKRLRTVILAPTRVVAAEMEEALRGLPVRYMTTAVNVTHSGTEIVDLMCHATFTSRLLQ

NC_002640|Dengue LKRRLRTLILAPTRVVAAEMEEALRGLPIRYQTPAVKSEHTGREIVDLMCHATFTTRLLS

NC_001563|West_Nile INKRLRTAVLAPTRVVAAEMSEALRGLPIRYQTSAVHREHSGNEIVDVMCHATLTHRLMS

NC_002031|Yellow_fever ARRRLRTLVLAPTRVVLSEMKEAFHGLDVKFHTQAFSAHGSGREVIDAMCHATLTYRMLE

NC_001437|Japanese_enceph IQQRLRTAVLAPTRVVAAEMAEALRGLPVRYQTSAVQREHQGNEIVDVMCHATLTHRLMS

NC_006551|Usutu_virus IQRRLRTAVLAPTRVVAAEMAEALKGLPVRYLTPAVNREHSGTEIVDVMCHATLTHRLMS

NC_007580|St_Louis IQKRLRTAVLAPTRVVACEIAEALKGLPIRYLTPAVRNEHQGNEIVDVMCHATLTQKLLT

NC_000943|Murray_Valley_enceph IQKRLRTAVLAPTRVVAAEMAEALRGLPVRYLTPAVQREHSGNEIVDVMCHATLTHRLMS

..**** :******* .*: **:.** :.: * *. * *::* *****:* .::

NC_012532|Zika PIRVPNYNLNIMDEAHFTDPSSIAARGYISTRVEMGEAAAIFMTATPPGTRDAFPDSNSP

NC_002640|Dengue STRVPNYNLIVMDEAHFTDPSSVAARGYISTRVEMGEAAAIFMTATPPGATDPFPQSNSP

NC_001563|West_Nile PHRVPNYNLFIMDEAHFTDPASIAARGYIATKVELGEAAAIFMTATPPGTSDPFPESNAP

NC_002031|Yellow_fever PTRVVNWEVIIMDEAHFLDPASIAARGWAAHRARANESATILMTATPPGTSDEFPHSNGE

NC_001437|Japanese_enceph PNRVPNYNLFVMDEAHFTDPASIAARGYIATKVELGEAAAIFMTATPPGTTDPFPDSNAP

NC_006551|Usutu_virus PLRAPNYNLFVMDEAHFTDPASIAARGYIATKVELGEAAAIFMTATPPGTHDPFPDTNAP

NC_007580|St_Louis PTRVPNYQVYIMDEAHFIDPASIAARGYISTKVELGEAAAIFMTATPPGTNDPFPDSNSP

NC_000943|Murray_Valley_enceph PLRVPNYNLFVMDEAHFTDPASIAARGYIATRVEAGEAAAIFMTATPPGTSDPFPDTNSP

. *. *::: :****** **:*:****: : .. .*:*:*:*******: * ** :*.

NC_012532|Zika IMDTEVEVPERAWSSGFDWVTDHSGKTVWFVPSVRNGNEIAACLTKAGKRVIQLSRKTFE

NC_002640|Dengue IEDIEREIPERSWNTGFDWITDYQGKTVWFVPSIKAGNDIANCLRKSGKKVIQLSRKTFD

NC_001563|West_Nile ISDMQTEIPDRAWNTGYEWITEYVGKTVWFVPSVKMGNEIALCLQRAGKKVIQLNRKSYE

NC_002031|Yellow_fever IEDVQTDIPSEPWNTGHDWILADKRPTAWFLPSIRAANVMAASLRKAGKSVVVLNRKTFE

NC_001437|Japanese_enceph IHDLQDEIPDRAWSSGYEWITEYAGKTVWFVASVKMGNEIAMCLQRAGKKVIQLNRKSYD

NC_006551|Usutu_virus VTDIQAEVPDRAWSSGFEWITEYTGKTVWFVASVKMGNEIAQCLQRAGKKVIQLNRKSYD

NC_007580|St_Louis ILDVEAQVPDKAWSTGYEWITNFTGRTVWFVPSVKSGNEIAICLQKAGKRVIQLNRKSFD

NC_000943|Murray_Valley_enceph VHDVSSEIPDRAWSSGFEWITDYAGKTVWFVASVKMSNEIAQCLQRAGKRVIQLNRKSYD

: * . ::*. .*.:*.:*: *.**:.*:. .* :* .* .:** *: *.**:::

NC_012532|Zika TEFQKTKNQEWDFVITTDISEMGANFKADRVIDSRRCLKPVILD--GERVILAGPMPVTH

NC_002640|Dengue TEYPKTKLTDWDFVVTTDISEMGANFRAGRVIDPRRCLKPVILPDGPERVILAGPIPVTP

NC_001563|West_Nile TEYPKCKNDDWDFVITTDISEMGANFKASRVIDSRKSVKPTIIEEGDGRVILGEPSAITA

NC_002031|Yellow_fever REYPTIKQKKPDFILATDIAEMGANLCVERVLDCRTAFKPVLVDE-GRKVAIKGPLRISA

NC_001437|Japanese_enceph TEYPKCKNGDWDFVITTDISEMGANFGASRVIDCRKSVKPTILEEGEGRVILGNPSPITS

NC_006551|Usutu_virus TEYPKCKNGDWDFVITTDISEMGANFGASRVIDCRKSVKPTILEEGEGRVILSNPSPITS

NC_007580|St_Louis TEYPKTKNNEWDFVVTTDISEMGANFGAHRVIDSRKCVKPVILED-DDRVILNGPMAITS

NC_000943|Murray_Valley_enceph TEYPKCKNGDWDFVITTDISEMGANFGASRVIDCRKSVKPTILDEGEGRVILSVPSAITS

*: . * . **:::***:*****: . **:* * ..**.:: .* : * ::

NC_012532|Zika ASAAQRRGRIGRNPNKPGDEYMYGGGCAETDEGHAHWLEARMLLDNIYLQDGLIASLYRP

NC_002640|Dengue ASAAQRRGRIGRNPAQEDDQYVFSGDPLKNDEDHAHWTEAKMLLDNIYTPEGIIPTLFGP

NC_001563|West_Nile ASAAQRRGRIGRNPSQVGDEYCYGGHTNEDDSNFAHWTEARIMLDNINMPNGLVAQLYQP

NC_002031|Yellow_fever SSAAQRRGRIGRNPNRDGDSYYYSEPTSENNAHHVCWLEASMLLDNMEVRGGMVAPLYGV

NC_001437|Japanese_enceph ASAAQRRGRVGRNPNQVGDEYHYGGATSEDDSNLAHWTEAKIMLDNIHMPNGLVAQLYGP

NC_006551|Usutu_virus ASAAQRRGRVGRNPSQIGDEYHYGGGTSEDDTIAAHWTEAKIMLDNIHLPNGLVAQMYGP

NC_007580|St_Louis ASAAQRRGRIGRNPSQIGDEYHYGGATNEDDHDLANWTEAKILLDNIYLPNGLVAQMYQP

NC_000943|Murray_Valley_enceph ASAAQRRGRVGRNPSQIGDEYHYGGGTSEDDTMLAHWTEAKILLDNIHLPNGLVAQLYGP

:********:**** . .*.* :. : : . * ** ::***: *::. ::

NC_012532|Zika EADKVAAIEGEFKLRTEQRKTFVELMKRGDLPVWLAYQVASAGITYTDRRWCFDGTTNNT

NC_002640|Dengue EREKTQAIDGEFRLRGEQRKTFVELMRRGDLPVWLSYKVASAGISYEDREWCFTGERNNQ

NC_001563|West_Nile EREKVYTMDGEYRLRGEERKNFLEFLRTADLPVWLAYKVAAAGISYHDRKWCFDGPRTNT

NC_002031|Yellow_fever EGTKTPVSPGEMRLRDDQRKVFRELVRNCDLPVWLSWQVAKAGLKTNDRKWCFEGPEEHE

NC_001437|Japanese_enceph EREKAFTMDGEYRLRGEEKKNFLELLRTADLPVWLAYKVASNGIQYTDRRWCFDGPRTNA

NC_006551|Usutu_virus ERDKAFTMDGEYRLRGEERKTFLELLRTADLPVWLAYKVASNGIQYTDRKWCFDGPRSNI

NC_007580|St_Louis ERDKVFTMDGEFRLRGEERKNFVELMRNGDLPVWLAYKVASNGHSYQDRSWCFTGQTNNT

NC_000943|Murray_Valley_enceph ERDKTYTMDGEYRLRGEERKTFLELIKTADLPVWLAYKVASNGIQYNDRKWCFDGPRSNI

* *. . ** .** ::.* * *::. ******:::** * ** *** * :

Mature peptide NS3-|-NS4a

NC_012532|Zika IMEDSVP-AEVWTKYGEKRVLKPRWMDARVCSDHAALKSFKEF**AAGKR**|**GAALG**VMEALGT

NC_002640|Dengue ILEENME-VEIWTREGEKKKLRPRWLDARVYADPMALKDFKEF**ASGRK**|**SITLD**ILTEIAS

NC_001563|West_Nile ILEDNNE-VEVITKLGERKILRPRWADARVYSDHQALKSFKDF**ASGKR**|**-SQIGL**VEVLGR

NC_002031|Yellow_fever ILNDSGETVKCRAPGGAKKPLRPRWCDERVSSDQSALSEFIKF**AEGRR**|**-GAAEV**LVVLSE

NC_001437|Japanese_enceph ILEDNTE-VEIVTRMGERKILKPRWLDARVYADHQALKWFKDF**AAGKR**|**-SAISF**IEVLGR

NC_006551|Usutu_virus ILEDNNE-VEIVTRTGERKMLKPRWLDARVYADHQSLKWFKDF**AAGKR**|**-SAVGF**LEVLGR

NC_007580|St_Louis ILEDNNE-VEVFTKTGDRKILRPKWMDARVCCDYQALKSFKEF**AAGKR**|**-SALGM**MEVMGR

NC_000943|Murray_Valley_enceph ILEDNNE-VEIITRIGERKVLKPRWLDARVYSDHQSLKWFKDF**AAGKR**|**-SAIGF**FEVLGR

*:::. .: : * .. *.*.* * ** .* :*. * .** *..| .. :.

NC_012532|Zika LPGHMTERFQEAIDNLAVLMRAETGSRPYKAAAAQLPETLETIMLLGLLGTVSLGIFFVL

NC_002640|Dengue LPTYLSSRAKLALDNIVMLHTTERGGRAYQHALNELPESLETLMLVALLGAMTAGIFLFF

NC_001563|West_Nile MPEHFMVKTWEALDTMYVVATAEKGGRAHRMALEELPDALQTIVLIALLSVMSLGVFFLL

NC_002031|Yellow_fever LPDFLAKKGGEAMDTISVFLHSEEGSRAYRNALSMMPEAMTIVMLFILAGLLTSGMVIFF

NC_001437|Japanese_enceph MPEHFMGKTREALDTMYLVATAEKGGKAHRMALEELPDALETITLIVAITVMTGGFFLLM

NC_006551|Usutu_virus MPEHFAGKTREAFDTMYLVATAEKGGKAHRMALEELPDALETITLIVALAVMTAGVFLLL

NC_007580|St_Louis MPNHFWEKTVAAADTLYLLGTSEANSRAHKEALAELPDSLETLLLIGMLCVMSMGTFIFL

NC_000943|Murray_Valley_enceph MPEHFAGKTREALDTMYLVATSEKGGKAHRMALEELPDALETITLIAALGVMTAGFFLLM

:* .: . * *.: :. :* ....:. * :*::: : *. :: * .:.:

Mature peptide NS4a-|-2K

NC_012532|Zika MRNKGIGKMGFGMVTLGASAWLMWLSEIEPARIACVLIVVFLLLVVLIPE**PEKQR**|**SPQDN**

NC_002640|Dengue MQGKGIGKLSMGLITIAVASGLLWVAEIQPQWIAASIILEFFLMVLLIPE**PEKQR**|**TPQDN**

NC_001563|West_Nile MQRKGIGKIGLGGVILGAATFFCWMAEVPGTKIAGMLLLSLLLMIVLIPE**PEKQR**|**SQTDN**

NC_002031|Yellow_fever MSPKGISRMSMAMGTMAGCGYLMFLGGVKPTHISYVMLIFFVLMVVVIPE**PGQQR**|**SIQDN**

NC_001437|Japanese_enceph MQRKGIGKMGLGALVLTLATFFLWAAEVPGTKIAGTLLIALLLMVVLIPE**PEKQR**|**SQTDN**

NC_006551|Usutu_virus VQRRGIGKLGLGGMVLGLATFFLWMADVSGTKIAGTLLLALLMMIVLIPE**PEKQR**|**SQTDN**

NC_007580|St_Louis MNRKGVGKMGLGAFVMTLATALLWAAEVPGTQIAGVLLIVFLLMIVLIPE**PEKQR**|**SQTDN**

NC_000943|Murray_Valley_enceph MQRKGIGKLGLGALVLVVATFFLWMSDVSGTKIAGVLLLALLMMVVLIPE**PEKQR**|**SQTDN**

: .*:..:.:. : . : : . : .*: ::: :.:::::**** :**|: **

Mature peptide 2K-|-NS4b

NC_012532|Zika QMAIIIMVAVGLL**GLITA**|**NELGW**LERTKNDIAHLMGRREE--GATMGFSM----DIDLRP

NC_002640|Dengue QLIYVILTILTII**GLIAA**|**NEMGL**IEKTKTDFGFYQVKTETT-----------ILDVDLRP

NC_001563|West_Nile QLAVFLICVLTLV**GAVAA**|**NEMGW**LDKTKNDIGSLLGHRPEARETTLGVESF---LLDLRP

NC_002031|Yellow_fever QVAYLIIGILTLV**SAVAA**|**NELGM**LEKTKED---LFGKKNLI---PSSASPWSWPDLDLKP

NC_001437|Japanese_enceph QLAVFLICVLTVV**GVVAA**|**NEYGM**LEKTKADLKSMFVGKTQA-SGLTGLPSM---ALDLRP

NC_006551|Usutu_virus QLAVFLICVLLVV**GVVAA**|**NEYGM**LERTKSDLGKIFSSTRQP-QSALPLPSMNALALDLRP

NC_007580|St_Louis QLAVFLICIMTLM**GVVAA**|**NEMGL**LEKTKSDIAKLFGSQPGSVGFATRTTPWDI-SLDIKP

NC_000943|Murray_Valley_enceph QLAVFLICVLLVV**GLVAA**|**NEYGM**LERTKTDIRNLFGKSLIE-ENEVHIPPFDFFTLDLKP

*: .:: : ::. ::*|** * ::.** * :*:.*

NC_012532|Zika ASAWAIYAALTTLITPAVQHAVTTSYNNYSLMAMATQAGVLFGMGKGMPFMHGDLGVPLL

NC_002640|Dengue ASAWTLYAVATTILTPMLRHTIENTSANLSLAAIANQAAVLMGLGKGWPLHRMDLGVPLL

NC_001563|West_Nile ATAWSLYAVTTAVLTPLLKHLITSDYINTSLTSINVQASALFTLARGFPFVDVGVSALLL

NC_002031|Yellow_fever GAAWTVYVGIVTMLSPMLHHWIKVEYGNLSLSGIAQSASVLSFMDKGIPFMKMNISVIML

NC_001437|Japanese_enceph ATAWALYGGSTVVLTPLLKHLITSEYVTTSLASINSQAGSLFVLPRGVPFTDLDLTVGLV

NC_006551|Usutu_virus ATAWALYGGSTVVLTPLIKHLVTSEYITTSLASISAQAGSLFNLPRGLPFTELDFTVVLV

NC_007580|St_Louis ATAWALYAAATMVMTPLIKHLITTQYVNFSLTAIASQAGVLLGLTNGMPFTAMDLSVPLL

NC_000943|Murray_Valley_enceph ATAWALYGGSTVVLTPLIKHLVTSQYVTTSLASINAQAGSLFTLPKGIPFTDFDLSVALV

.:**::* . :::* :.* : . ** .: .*. * : .* *: .. . ::

NC_012532|Zika MMGCYSQLTPLTLIVAIILLVAHYMYLIPGLQAAAARAAQKRTAAGIMKNPVVDGIVVTD

NC_002640|Dengue AMGCYSQVNPTTLTASLVMLLVHYAIIGPGLQAKATREAQKRTAAGIMKNPTVDGITVID

NC_001563|West_Nile AVGCWGQVTLTVTVTAAALLFCHYAYMVPGWQAEAMRSAQRRTAAGIMKNVVVDGIVATD

NC_002031|Yellow_fever LVSGWNSITVMPLLCGIGCAMLHWSLILPGIKAQQSKLAQRRVFHGVAENPVVDGNPTVD

NC_001437|Japanese_enceph FLGCWGQITLTTFLTAMVLATLHYGYMLPGWQAEALRAAQRRTAAGIMKNAVVDGMVATD

NC_006551|Usutu_virus FLGCWGQVSLTTLITAAALATLHYGYMLPGWQAEALRAAQRRTAAGIMKNAVVDGLVATD

NC_007580|St_Louis VLGCWNQMTLPSLAVAVMLLAIHYAFMIPGWQAEAMRAAQRRTAAGIMKNAVVDGIVATD

NC_000943|Murray_Valley_enceph FLGCWGQVTLTTLIMATILVTLHYGYLLPGWQAEALRAAQKRTAAGIMKNAVVDGIVATD

:. :..:. . *: : ** :* . **.*. *: :* .*** . *

NC_012532|Zika IDTMT-IDPQVEKKMGQVLLIAVAISSAVLLRTAWGWGEAGALITAATSTLWEGSPNKYW

NC_002640|Dengue LEPIS-YDPKFEKQLGQVMLLVLCAGQLLLMRTTWAFCEVLTLATGPILTLWEGNPGRFW

NC_001563|West_Nile VPELERTTPVMQKKVGQIILILVSMAAVVVNPSVRTVREAGILTTAAAVTLWENGASSVW

NC_002031|Yellow_fever IEEAPEMPALYEKKLALYLLLALSLASVAMCRTPFSLAEGIVLASAALGPLIEGNTSLLW

NC_001437|Japanese_enceph VPELERTTPLMQKKVGQVLLIGVSVAAFLVNPNVTTVREAGVLVTAATLTLWDNGASAVW

NC_006551|Usutu_virus VPELERTTPLMQKKVGQILLIGVSAAALLVNPCVTTVREAGILISAALLTLWDNGAIAVW

NC_007580|St_Louis IPDLSPATPMTEKKMGQILLIAAAVLAVLVRPGICSIKEFGVLGSAALVTLIEGTAGVVW

NC_000943|Murray_Valley_enceph VPELERTTPQMQKRLGQILLVLASVAAVCVNPRITTIREAGILCTAAALTLWDNNASAAW

: . :*.:. :*: . : * * :.. .* :. . *

Mature peptide NS4b-|-NS5

NC_012532|Zika NSSTATSLCNIFRGSYLAGASLIYTVTRNAG**LVK-RR**|**GGGT-G**ETLGEKWKARLNQMSAL

NC_002640|Dengue NTTIAVSTANIFRGSYLAGAGLAFSLIKNA**QTP--RR**|**GTGTT**GETLGEKWKRQLNSLDRK

NC_001563|West_Nile NATTAIGLCHIMRGGWLSCLSIMWTLIKNMEK**PGLKR**|**GGAK-G**RTLGEVWKERLNHMTKE

NC_002031|Yellow_fever NGPMAVSMTGVMRGNHYAFVGVMYNLWK---M**KTGRR**|**GSAN-G**KTLGEVWKRELNLLDKR

NC_001437|Japanese_enceph NSTTATGLCHVMRGSYLAGGSIAWTLIKNADK**PSLKR**|**GRPG-G**RTLGEQWKEKLNAMSRE

NC_006551|Usutu_virus NSTTATGLCHVIRGNWLAGASIAWTLIKNADK**PACKR**|**GRPG-G**RTLGEQWKEKLNGLSKE

NC_007580|St_Louis NCTTAVGLCNLMRGGWLAGMSITWTVYKNVDK**PKGKR**|**GGGK-G**ATLGEIWKSRLNQLTRA

NC_000943|Murray_Valley_enceph NSTTATGLCHVMRGSWIAGASIAWTLIKNAEK**PAFKR**|**GRAG-G**RTLGEQWKEKLNAMGKE

* . * . ::**. : .: :.: . .*|* * **** ** ** :

NC_012532|Zika EFYSYKKSGITEVCREEARRALKDGVATGGHAVSRGSAKIRWLEERGYLQPYGKVVDLGC

NC_002640|Dengue EFEEYKRSGILEVDRTEAKSALKDGSKIK-HAVSRGSSKIRWIVERGMVKPKGKVVDLGC

NC_001563|West_Nile EFTRYRKEAITEVDRSAAKHARREGNITGGHPVSRGTAKLRWLVERRFLEPVGKVVDLGC

NC_002031|Yellow_fever QFELYKRTDIVEVDRDTARRHLAEGKVDTGVAVSRGTAKLRWFHERGYVKLEGRVIDLGC

NC_001437|Japanese_enceph EFFKYRREAIIEVDRTEARRARRENNIVGGHPVSRGSAKLRWLVEKGFVSPIGKVIDLGC

NC_006551|Usutu_virus DFLKYRKEAITEVDRSAARKARRDGNKTGGHPVSRGSAKLRWMVERQFVKPIGKVVDLGC

NC_007580|St_Louis EFMAYRKDGIVEVDRAPARKARREGRLTGGHPVSRGSAKLRWITERGFVKPMGKVVDLGC

NC_000943|Murray_Valley_enceph EFFSYRKEAILEVDRTEARRARREGNKVGGHPVSRGTAKLRWLVERRFVQPIGKVVDLGC

:* *.. * ** * *. :. .****::*:**: *. :. *.*:****

NC_012532|Zika GRGGWSYYAATIRKVQEVRGYTKGGPGHEEPMLVQSYGWNIVRLKSGVDVFHMAAEPCDT

NC_002640|Dengue GRGGWSYYMATLKNVTEVKGYTKGGPGHEEPIPMATYGWNLVKLHSGVDVFYKPTEQVDT

NC_001563|West_Nile GRGGWCYYMATQKRVQEVKGYTKGGPGHEEPQLVQSYGWNIVTMKSGVDVFYRPSEASDT

NC_002031|Yellow_fever GRGGWCYYAAAQKEVSGVKGFTLGRDGHEKPMNVQSLGWNIITFKDKTDIHRLEPVKCDT

NC_001437|Japanese_enceph GRGGWSYYAATLKKVQEVRGYTKGGAGHEEPMLMQSYGRNLVSLKSGVDVFYKPSEPSDT

NC_006551|Usutu_virus GRGGWSYYAATLKGVQEVRGYTKGGPGHEEPMLMQSYGWNLVTMKSGVDVYYKPSEPCDT

NC_007580|St_Louis GRGGWSYYCATLKHVQEVKGFTKGGPGHEEPQLMQSYGWNLVHMKSGVDVFHKPAEPADT

NC_000943|Murray_Valley_enceph GRGGWSYYAATMKNVQEVRGYTKGGPGHEEPMLMQSYGWNIVTMKSGVDVFYKPSEISDT

*****.** *: . * *.*:* * ***:* : : *.*:: ::. .*:. . **

NC_012532|Zika LLCDIGESSSSPEVEETRTLRVLSMVGDWLEKRPGAFCIKVLCPYTSTMMETMERLQRRH

NC_002640|Dengue LLCDIGESSSNPTIEEGRTLRVLKMVEPWLSSKP-EFCIKVLNPYMPTVIEELEKLQRKH

NC_001563|West_Nile LLCDIGESSSSAEVEEHRTVRVLEMVEDWLHRGPKEFCIKVLCPYMPKVIEKMETLQRRY

NC_002031|Yellow_fever LLCDIGESSSSSVTEGERTVRVLDTVEKWLACGVDNFCVKVLAPYMPDVLEKLELLQRRF

NC_001437|Japanese_enceph LFCDIGESSPSPEVEEQRTLRVLEMTSDWLHRGPREFCIKVLCPYMPKVIEKMEVLQRRF

NC_006551|Usutu_virus LFCDIGESSSSAEVEEQRTLRILEMVSDWLQRGPREFCIKVLCPYMPRVMERLEVLQRRY

NC_007580|St_Louis VLCDIGESNPSCEVEEARTARVLDMVEEWLKKGATEFCIKVLCPYTPKIIEKLEKLQRKY

NC_000943|Murray_Valley_enceph LLCDIGESSPSAEIEEQRTLRILEMVSDWLSRGPKEFCIKILCPYMPKVIEKLESLQRRF

::******... * ** *:*. . ** **:*:* ** . ::* :* ***..

NC_012532|Zika GGGLVRVPLCRNSTHEMYWVSGAKSNIIKSVSTTSQLLLGRMDGPRRP-VKYEEDVNLGS

NC_002640|Dengue GGNLVRCPLSRNSTHEMYWVSGASGNIVSSVNTTSKMLLNRFTTRHRK-PTYEKDVDLGA

NC_001563|West_Nile GGGLIRNPLSRNSTHEMYWVSHASGNIVHSVNMTSQVLLGRMEKKTWKGPQFEEDVNLGS

NC_002031|Yellow_fever GGTVIRNPLSRNSTHEMYYVSGARSNVTFTVNQTSRLLMRRMRRPTGK-VTLEADVILPI

NC_001437|Japanese_enceph GGGLVRLPLSRNSNHEMYWVSGAAGNVVHAVNMTSQVLLGRMDRTVWRGPKYEEDVNLGS

NC_006551|Usutu_virus GGGLVRVPLSRNSNHEMYWVSGAAGNIVHAVNMTSQVLIGRMEKRTWHGPKYEEDVNLGS

NC_007580|St_Louis GGGLVRVPLSRNSTHEMYWVSGAAGNIIHAVSMTSQVLMGRMDKQNRSGPRYEEDVNLGS

NC_000943|Murray_Valley_enceph GGGLVRVPLSRNSNHEMYWVSGASGNIVHAVNMTSQVLIGRMDKKIWKGPKYEEDVNLGS

** ::* **.***.****:** * .*: :*. **.:*: *: * ** *

NC_012532|Zika GTRAVASCAEAPNMKIIGRRIERIRNEHAETWFLDENHPYRTWAYHGSYEAPTQGSASSL

NC_002640|Dengue GTRSVSTETEKPDMTIIGRRLQRLQEEHKETWHYDQENPYRTWAYHGSYEAPSTGSASSM

NC_001563|West_Nile GTRAVGKPLLNSDTSKIKNRIERLKKEYSSTWHQDANHPYRTWNYHGSYEVKPTGSASSL

NC_002031|Yellow_fever GTRSVETDKGPLDKEAIEERVERIKSEYMTSWFYDNDNPYRTWHYCGSYVTKTSGSAASM

NC_001437|Japanese_enceph GTRAVGKGEVHSNQEKIKKRIQKLKEEFATTWHKDPEHPYRTWTYHGSYEVKATGSASSL

NC_006551|Usutu_virus GTRAVGKPQPHTNQEKIKARIQRLKEEYAATWHHDKDHPYRTWTYHGSYEVKPTGSASSL

NC_007580|St_Louis GTRSVGKLTEKPDPRKVGERIRRLREEYQQTWTYDHNNPYRTWNYHGSYEVKPTGSASSM

NC_000943|Murray_Valley_enceph GTRAVGKGVQHTDYKRIKSRIEKLKEEYAATWHTDDNHPYRTWTYHGSYEVKPSGSASTL

***:* . : : *: .:..*. :* * ::***** * *** . . ***:::

NC_012532|Zika VNGVVRLLSKPWDVVTGVTGIAMTDTTPYGQQRVFKEKVDTRVPDPQEGTRQVMNIVSSW

NC_002640|Dengue VNGVVKLLTKPWDVIPMVTQLAMTDTTPFGQQRVFKEKVDTRTPQPKPGTRMVMTTTANW

NC_001563|West_Nile VNGVVRLLSKPWDTITNVTTMAMTDTTPFGQQRVFKEKVDTKAPEPPEGVKYVLNETTNW

NC_002031|Yellow_fever VNGVIKILTYPWDRIEEVTRMAMTDTTPFGQQRVFKEKVDTRAKDPPAGTRKIMKVVNRW

NC_001437|Japanese_enceph VNGVVKLMSKPWDAIANVTTMAMTDTTPFGQQRVFKEKVDTKAPEPPAGAKEVLNETTNW

NC_006551|Usutu_virus VNGVVRLMSKPWDAILNVTTMAMTDTTPFGQQRVFKEKVDTKAPEPPSGVREVMDETTNW

NC_007580|St_Louis VNGVVRLLSKPWDMITNVTTMAMTDTTPFGQQRVFKEKVDTKAPEPPLGVAQIMDVTTDW

NC_000943|Murray_Valley_enceph VNGVVRLLSKPWDAITGVTTMAMTDTTPFGQQRVFKEKVDTKAPEPPQGVKTVMDETTNW

****:.::: *** : ** :*******:************.. :* *. :: . *

NC_012532|Zika LWKELGKRKRPRVCTKEEFINKVRSNAALGAIFEEEKEWKTAVEAVNDPRFWALVDRERE

NC_002640|Dengue LWALLGKKKNPRLCTREEFISKVRSNAAIGAVFQEEQGWTSASEAVNDSRFWELVDKERA

NC_001563|West_Nile LWAFLARDKKPRMCSREEFIGKVNSNAALGAMFEEQNQWKNAREAVEDPKFWEMVDEERE

NC_002031|Yellow_fever LFRHLAREKNPRLCTKEEFIAKVRSHAAIGAYLEEQEQWKTANEAVQDPKFWELVDEERK

NC_001437|Japanese_enceph LWAHLSREKRPRLCTKEEFIKKVNSNAALGAVFAEQNQWSTAREAVDDPRFWEMVDEERE

NC_006551|Usutu_virus LWAFLAREKKPRLCTREEFKRKVNSNAALGAMFEEQNQWSSAREAVEDPRFWEMVDEERE

NC_007580|St_Louis LWDFVAREKKPRVCTPEEFKAKVNSHAALGAMFEEQNQWSSAREAVEDPKFWEMVDEERE

NC_000943|Murray_Valley_enceph LWAYLARNKKARLCTREEFVKKVNSHAALGAMFEEQNQWKNAREAVEDPKFWEMVDEERE

*: :.. *..*:*: *** **.*:**:** : *:: *..* ***:*..** :** **

NC_012532|Zika HHLRGECHSCVYNMMGKREKKQGEFGKAKGSRAIWYMWLGARFLEFEALGFLNEDHWMGR

NC_002640|Dengue LHQEGKCESCVYNMMGKREKKLGEFGRAKGSRAIWYMWLGARFLEFEALGFLNEDHWFGR

NC_001563|West_Nile AHLRGECNTCIYNMMGKREKKPGEFGKAKGSRAIWFMWLGARFLEFEALGFLNEDHWLGR

NC_002031|Yellow_fever LHQQGRCRTCVYNMMGKREKKLSEFGKAKGSRAIWYMWLGARYLEFEALGFLNEDHWASR

NC_001437|Japanese_enceph NHLRGECHTCIYNMMGKREKKPGEFGKAKGSRAIWFMWLGARYLEFEALGFLNEDHWLSR

NC_006551|Usutu_virus NHLKGECHTCIYNMMGKREKKLGEFGKAKGSRAIWFMWLGARFLEFEALGFLNEDHWLGR

NC_007580|St_Louis AHLKGECHTCIYNMMGKREKKTGEFGKAKGSRAIWYMWLGARFLEFEALGFLNEDHWMSR

NC_000943|Murray_Valley_enceph CHLRGECRTCIYNMMGKREKKPGEFGKAKGSRAIWFMWLGARFLEFEALGFLNEDHWMSR

* * * :*:********** .***.********:******:************** .*

NC_012532|Zika ENSGGGVEGLGLQRLGYILEEMNRAPGGKMYADDTAGWDTRISKFDLENEALITNQMEEG

NC_002640|Dengue ENSWSGVEGEGLHRLGYILEEIDKKDGDLMYADDTAGWDTRITEDDLQNEELITEQMAPH

NC_001563|West_Nile KNSGGGVEGLGLQKLGYILKEVGTKPGGKVYADDTAGWDTRITKADLENEAKVLELLDGE

NC_002031|Yellow_fever ENSGGGVEGIGLQYLGYVIRDLAAMDGGGFYADDTAGWDTRITEADLDDEQEILNYMSPH

NC_001437|Japanese_enceph ENSGGGVEGSGVQKLGYILRDIAGKQGGKMYADDTAGWDTRITRTDLENEAKVLELLDGE

NC_006551|Usutu_virus KNSGGGVEGLGVQKLGYILREMSHHSGGKMYADDTAGWDTRITRADLDNEAKVLELMEGE

NC_007580|St_Louis ENSYGGVEGKGLQKLGYILQEISQIPGGKMYADDTAGWDTRITKEDLKNEAKITKRMEER

NC_000943|Murray_Valley_enceph ENSGGGVEGAGIQKLGYILRDVAQKPGGKIYADDTAGWDTRITQADLENEAKVLELMEGE

:** .**** *:: ***:: :: *. .************: **.:* : : :

NC_012532|Zika HRTLALAVIKYTYQNKVVKVLRPAEGGKTVMDIISRQDQRGSGQVVTYALNTFTNLVVQL

NC_002640|Dengue HKILAKAIFKLTYQNKVVKVLRPTPRG-AVMDIISRKDQRGSGQVGTYGLNTFTNMEVQL

NC_001563|West_Nile HRRLARSIIELTYRHKVVKVMRPAADGKTVMDVISREDQRGSGQVVTYALNTFTNLAVQL

NC_002031|Yellow_fever HKKLAQAVMEMTYKNKVVKVLRPAPGGKAYMDVISRRDQRGSGQVVTYALNTITNLKVQL

NC_001437|Japanese_enceph HRMLARAIIELTYRHKVVKVMRPAAEGKTVMDVISREDQRGSGQVVTYALNTFTNIAVQL

NC_006551|Usutu_virus HRQLARAIIELTYKHKVVKVMRPGTDGKTVMDVISREDQRGSGQVVTYALNTFTNIAVQL

NC_007580|St_Louis HRKLAEAIIDLTYRHKVVKVMRPGPDGKTYMDVISREDQRGSGQVVTYALNTFTNLAVQL

NC_000943|Murray_Valley_enceph QRTLARAIIELTYRHKVVKVMRPAAGGKTVMDVISREDQRGSGQVVTYALNTFTNIAVQL

:. ** :::. **.:*****:** * : **:*** ******** **.***:**: ***

NC_012532|Zika IRNMEAEEVLEMQDLWLLRKP--EKVTRWLQSNGWDRLKRMAVSGDDCVVKPIDDRFAHA

NC_002640|Dengue IRQMEAEGVITQDDMQN-PKGLKERVEKWLKECGVDRLKRMAISGDDCVVKPLDERFGTS

NC_001563|West_Nile VRMMEGEGVIGPDDVEKLGKGKGPKVRTWLFENGEERLSRMAVSGDDCVVKPLDDRFATS

NC_002031|Yellow_fever IRMAEAEMVIHHQHVQDCDESVLTRLEAWLTEHGCDRLKRMAVSGDDCVVRPIDDRFGLA

NC_001437|Japanese_enceph VRLMEAEGVIGPQHLEQLPRKTKIAVRTWLFENGEERVTRMAISGDDCVVKPLDDRFATA

NC_006551|Usutu_virus IRLMEAEGVIGQEHLESLPRKTKYAVRTWLFENGEERVTRMAVSGDDCVVKPLDDRFANA

NC_007580|St_Louis IRCMEAEGVVDEDDITRVRLGRLAKAVEWLRKNGPERLSRMAVSGDDCVVKPIDDRFATA

NC_000943|Murray_Valley_enceph VRLMEAEAVIGPDDIESIERKKKFAVRTWLFENAEERVQRMAVSGDDCVVKPLDDRFSTA

:* *.* *: : : ** . . :*: ***:*******.*:*:**. :

NC_012532|Zika LRFLNDMGKVRKDTQEWKPSTGWSNWEEVPFCSHHFNKLYLKDGRSIVVPCRHQDELIGR

NC_002640|Dengue LLFLNDMGKVRKDIPQWEPSKGWKNWQEVPFCSHHFHKIFMKDGRSLVVPCRNQDELIGR

NC_001563|West_Nile LHFLNAMSKVRKDIQEWKPSTGWYDWQQVPFCSNHFTELIMKDGRTLVVPCRGQDELIGR

NC_002031|Yellow_fever LSHLNAMSKVRKDISEWQPSKGWNDWENVPFCSHHFHELQLKDGRRIVVPCREQDELIGR

NC_001437|Japanese_enceph LHFLNAMSKVRKDIQEWKPSHGWHDWQQVPFCSNHFQEIVMKDGRSIVVPCRGQDELIGR

NC_006551|Usutu_virus LHFLNSMSKVRKDVPEWKPSSGWHDWQQVPFCSNHFQELIMKDGRTLVVPCRGQDELIGR

NC_007580|St_Louis LHFLNNMSKIRKDIQEWKPSTGWHNWQEVPFCSHHFNELMLKDGRTIVVPCRSQDELIGR

NC_000943|Murray_Valley_enceph LHFLNAMSKVRKDIQEWKPSQGWYDWQQVPFCSNHFQEVIMKDGRTLVVPCRGQDELIGR

* .** *.*:*** :*:** ** :*::*****:** :: :**** :***** *******

NC_012532|Zika ARVSPGAGWSIRETACLAKSYAQMWQLLYFHRRDLRLMANAICSAVPVDWVPTGRTTWSI

NC_002640|Dengue ARISQGAGWSLRETACLGKAYAQMWSLMYFHRRDLRLASMAICSAVPTEWFPTSRTTWSI

NC_001563|West_Nile ARISPGAGWNVRDTACLAKSYAQMWLLLYFHRRDLRLMANAICSAVPANWVPTGRTTWSI

NC_002031|Yellow_fever GRVSPGNGWMIKETACLSKAYANMWSLMYFHKRDMRLLSLAVSSAVPTSWVPQGRTTWSI

NC_001437|Japanese_enceph ARISPGAGWNVKDTACLAKAYAQMWLLLYFHRRDLRLMANAICSAVPVDWVPTGRTSWSI

NC_006551|Usutu_virus ARVSPGSGWNVRDTACLAKAYAQMWLLLYFHRRDLRLMANAICSAVPSNWVPTGRTSWSV

NC_007580|St_Louis ARISPGAGWNVKETACLSKSYAQMWLLMYFHRRDLRMMANAICSAVPVNWVPTGRTTWSI

NC_000943|Murray_Valley_enceph ARISPGSGWNVRDTACLAKAYAQMWLVLYFHRRDLRLMANAICSSVPVDWVPTGRTTWSI

.*:* * ** :.:****.*:**:** ::***.**:*: : *:.*:** .*.* .**:**:

NC_012532|Zika HGKGEWMTTEDMLMVWNRVWIEENDHMEDKTPVTKWTDIPYLGKREDLWCGSLIGHRPRT

NC_002640|Dengue HAHHQWMTTEDMLKVWNRVWIEDNPNMTDKTPVHSWEDIPYLGKREDLWCGSLIGLSSRA

NC_001563|West_Nile HAKGEWMTTEDMLAVWNRVWIEENEWMEDKTPVERWSDVPYSGKREDIWCGSLIGTRTRA

NC_002031|Yellow_fever HGKGEWMTTEDMLEVWNRVWITNNPHMQDKTMVKKWRDVPYLTKRQDKLCGSLIGMTNRA

NC_001437|Japanese_enceph HSKGEWMTTEDMLQVWNRVWIEENEWMMDKTPITSWTDVPYVGKREDIWCGSLIGTRSRA

NC_006551|Usutu_virus HATGEWMTTDDMLEVWNKVWIQDNEWMLDKTPVQSWTDIPYTGKREDIWCGSLIGTRTRA

NC_007580|St_Louis HGKGEWMTTEDMLSVWNRVWIEENEYMKDKTPLAAWNDIPYLGKREDIWCGSLIGTRTRA

NC_000943|Murray_Valley_enceph HGKGEWMTTEDMLSVWNRVWILENEWMEDKTTVSDWTEVPYVGKREDIWCGSLIGTRTRA

*. :****:*** ***.*** :* * *** : * ::** **:* ****** *:

Mature peptide NS5-

NC_012532|Zika TWAENIKDTVNMVRRIIGDEEKYMDYLSTQVRYL-GEEGS**TPGVL**

NC_002640|Dengue TWAKNIHTAITQVRNLIGKEE-YVDYMPVMKRYS--APSE**SEGVL**

NC_001563|West_Nile TWAENIHVAINQVRSVIGEEK-YVDYMSSLRRYE-DTIVV**EDTVL**

NC_002031|Yellow_fever TWASHIHLVIHRIRTLIGQEK-YTDYLTVMDRYSVDADLQ**LGELI**

NC_001437|Japanese_enceph TWAENIYAAINQVRAVIGKEN-YVDYMTSLRRYE-DVLIQ**EDRVI**

NC_006551|Usutu_virus TWAENIYAAINQVRAIIGQEK-YRDYMLSLRRYE-EVNVQ**EDRVL**

NC_007580|St_Louis TWAENIYAPIMQIRNLIGEEE-YRDYMVAQNRFGREETHV**VGGVL**

NC_000943|Murray_Valley_enceph TWAENIYAAINQVRSVIGKEK-YVDYVQSLRRYE-ETHVS**EDRVL**

***.:* : :* :**.*: * **: *: ::

========= End of MSA ================

**Table B**. **Reference genome sequences for ZIKV genotype analysis**. The reference genomes for the genotype analysis were chosen from full-length ZIKV genomes plus several incomplete sequences that have been used in other studies [2,3,4]. The complete list of the reference ZIKV genomes are listed in Table B.

| **Genotype** | **ZIKV strain** | **NCBI accession** | **Year** | **Source country** | **Short sequence** |
| --- | --- | --- | --- | --- | --- |
| East African | ArB1362 | KF383115 | 1968 | Central African Rep | no |
| East African | ArD157995 | KF383118 | 2001 | Senegal | no |
| East African | ArD158084 | KF383119 | 2001 | Senegal | no |
| East African | ArD158095 | KF383121 |  |  | yes |
| East African | MR 766 | AY632535, NC_012532 | 1947 | Uganda | no |
| East African | MR766-NIID | LC002520 |  | Uganda | no |
| East African | MR 766 | DQ859059 |  | Uganda | no |
| East African | MR_766 | HQ234498 | 1947 | Uganda | no |
| East African | ARB13565 | KF268948 | 1976 | Central African Rep | no |
| East African | ARB15076 | KF268949 |  | Central African Rep | no |
| East African | ARB7701 | KF268950 |  | Central African Rep | no |
| West African | IbH_30656 | HQ234500 | 1968 | Nigeria | no |
| West African | ArD_41519 | HQ234501 | 1984 | Senegal | no |
| West African | ArD7117 | KF383116 | 1968 | Senegal | no |
| West African | ArD128000 | KF383117 | 1997 | Senegal | no |
| Asian | P6-740 | HQ234499 | 1966 | Malaysia | no |
| Asian | FSM | EU545988 | 2007 | Yap Island | no |
| Asian | FSS13025 | JN860885 | 2010 | Cambodia | no |
| Asian | PLCal_ZV | KF993678 | 2013 | Thailand | no |
| Asian | CK-ISL 2014 | KJ634273 | 2014 | Cook Islands | yes |
| Asian | H/PF/2013 | KJ776791 | 2013 | French Polynesia | no |
| Asian | ZikaSPH2015 | KU321639 | 2015 | Brazil | no |
| Asian | BeH818995 | KU365777 | 2015 | Brazil | no |
| Asian | BeH819015 | KU365778 | 2015 | Brazil | no |
| Asian | BeH819966 | KU365779 | 2015 | Brazil | no |
| Asian | BeH815744 | KU365780 | 2015 | Brazil | no |
| Asian | Z1106033 | KU312312 | 2015 | Suriname | no |
| Asian | Z1106032 | KU312313 | 2015 | Suriname | yes |
| Asian | Z1106031 | KU312314 | 2015 | Suriname | yes |
| Asian | Z1106027 | KU312315 | 2015 | Suriname | yes |

**References**:

1. Edgar RC. MUSCLE: multiple sequence alignment with high accuracy and high throughput. Nucleic Acids Res. 2004; 32(5):1792-1797.

2. Faye O, Freire CCM, Iamarino A, et al. Molecular evolution of Zika virus during its emergence in the 20th century. PLOS Neg. Trop. Dis. 2014; 8:e2636.

3. Lanciotti R, Lambert A, Holodniy M, Saavedra S, Signor L. Phylogeny of Zika Virus in Western Hemisphere, 2015. Emerg Infect Dis. 2016;22(5):933-935. https://dx.doi.org/10.3201/eid2205.1600654.

4. Enfissi A, Codrington J, Roosblad J, et al. Zika virus genome from the Americas. Lancet*.* 2016; 387:227-228.
